# Phenotypic heterogeneity optimizes trade-offs during adaptive deployment of the type VI secretion system

**DOI:** 10.1371/journal.pbio.3003838

**Published:** 2026-06-04

**Authors:** Boris Taillefer, Florian Schattenberg, Thierry Doan, Susann Müller, Eric Cascales

**Affiliations:** 1 Laboratoire d’Ingénierie des Systèmes Macromoléculaires (LISM), Aix-Marseille University, CNRS, Marseille, France; 2 Department of Applied Microbial Ecology, Helmholtz Centre for Environmental Research - UFZ, Leipzig, Germany; Max Planck Institute for Terrestrial Microbiology: Max-Planck-Institut fur terrestrische Mikrobiologie, GERMANY

## Abstract

The type VI secretion system (T6SS) is a widespread nanoweapon deployed by bacteria to eliminate competitors in polymicrobial environments, allowing niche colonization or host invasion. Fluorescence microscopy recordings have shown that T6SS expression and/or activation is heterogeneous in clonal populations of many bacterial species. However, it is still unknown whether T6SS heterogeneity is genetically controlled or arises from stochastic processes and what its physiological relevance is. Here, we report that enteroaggregative *Escherichia coli* (EAEC) exhibits stable phenotypic heterogeneity in T6SS expression. Under iron-limiting conditions, the Sci1 T6SS is expressed in only a subset of the population, creating distinct ON and OFF subpopulations in a reversible, heritable, and epigenetically controlled equilibrium. This heterogeneity is governed by the interplay between the iron-responsive regulator Fur- and Dam-dependent DNA methylation at the *sci1* promoter. Mutations in Fur binding sites or GATC methylation motifs shift the population to homogeneous ON or OFF states, respectively. Functional analyses reveal that while ON cells mediate antibacterial activity, OFF cells buffer the population against lethal retaliatory responses from defensive T6SS⁺ competitors. Our results suggest that T6SS heterogeneity in EAEC represents a finely tuned attenuation strategy optimizing the trade-off between competitive killing and survival in hostile microbial communities. This work uncovers a novel layer of regulation in T6SS deployment and highlights phenotypic heterogeneity as an adaptive trait in interbacterial warfare.

## Abstract 2

The type VI secretion system (T6SS) is a widespread nanoweapon deployed by bacteria, but why is its deployment heterogeneous in many species? This study of T6SS in Escherichia coli shows that adopting distinct attacker "ON" and defender "OFF" roles optimizes survival, and is a key strategy in microbial warfare.

## Introduction

The type VI secretion system (T6SS) is a contractile nanoweapon that delivers effectors into neighboring cells via a spring-like injection mechanism [1]. By targeting both bacterial competitors and eukaryotic host cells, the T6SS plays a central role in microbial competition, polymicrobial community dynamics, and pathogenesis [2]. T6SS gene clusters are widespread in gram-negative bacteria inhabiting diverse ecological niches, including opportunistic respiratory pathogens, enteric pathogens and commensals, plant-associated symbionts and pathogens, and free-living soil and marine species [3,4]. Because T6SS activity can profoundly alter microbial interactions and host colonization, its expression is tightly regulated by a range of environmental cues [5,6]. T6SSs gene clusters respond to physical, chemical, and nutritional signals, including cell density, pH, temperature, salinity, viscosity, nutrient availability, host-derived stimuli or stresses, via quorum sensing, two-component systems, alternative sigma factors, or global regulators [5,6].

In enteroaggregative *Escherichia coli* (EAEC), the *sci1* T6SS gene cluster is controlled by iron availability through the ferric uptake regulator Fur, and by the DNA adenine methylase Dam [7]. The promoter of the *sci1* gene cluster (P*sci1*) is comprised of two Fur binding sites (proximal F1 and distal F2 boxes) and three GATC sites (G1, G2, and G3), with the F1 box and G1 site overlapping the transcriptional −10 element. This architecture establishes direct competition between Fur, Dam, and RNA polymerase for promoter occupancy in an iron-dependent manner: under iron-rich conditions, holo-Fur binds to the promoter, preventing G1 methylation and repressing *sci1* expression (OFF state). Conversely, under iron starvation, apo-Fur loses affinity for the promoter, allowing Dam-mediated methylation of G1 and favoring promoter activation and expression of the T6SS *sci1* gene cluster (ON state) [7].

Promoter architectures that couple transcription factor binding with DNA methylation can generate bistable gene expression states, producing epigenetic regulation and phenotypic heterogeneity [8]. Indeed, fluorescence microscopy using translational fusions to T6SS core components has suggested heterogeneous expression in EAEC [9–15]. T6SS heterogeneous expression or activation has also been observed in several other species [16–22]. Phenotypic heterogeneity refers to the emergence of distinct phenotypic states within a clonal population, driven by cell-to-cell variation in gene expression [8,23–25]. Such heterogeneity can arise from genetic (e.g., DNA rearrangements) or nongenetic (e.g., DNA methylation, feedback loops, and stochastic fluctuations) mechanisms. In *Yersinia pseudotuberculosis*, T6SS4 heterogeneous expression has been shown to be controlled by the heterogeneous expression of the gene encoding the RovC transcriptional regulator [21], whereas the *Pseudomonas aeruginosa* T6SS-H1 bistable expression is controlled by c-di-GMP levels via a yet unknown mechanism [22]. These observations suggest that phenotypic heterogeneity might be a recurring feature of T6SS regulation, although the underlying mechanisms can differ markedly between bacterial species.

Phenotypic heterogeneity can provide adaptive advantages through strategies such as bet-hedging and division of labor [26]. In bet-hedging, subpopulations are pre-adapted to unpredictable environmental changes, increasing the overall survival of the population despite the cost of expressing traits that may be disadvantageous under certain conditions. Examples include antimicrobial peptide resistance in a minority of *Photorhabdus laumondii* cells, which is essential for successful host invasion [27]. In division of labor, phenotypically distinct subpopulations accomplish specialized tasks independently that enhance group fitness [28]. The *P. aeruginosa* T6SS-H1 activity is inversely associated with expression of other virulence factors such as the T3SS, suggesting that T6SS-H1 heterogeneity can be embedded in broader physiological specialization programs [22]. Such a strategy is also observed in *Myxococcus xanthus* fruiting body formation [29] and in heterogeneous expression of the *Salmonella enterica* type III secretion system (T3SS), where ON cells enable host invasion while OFF cells divide more rapidly to sustain infection [30]. In this case, the ON/OFF subpopulation equilibrium is critical for pathogenicity since a slight change in the ratio reduces the infection success [31]. While an increasing number of studies have described phenotypic heterogeneity in various bacterial traits, the role and ecological impact of this phenomenon remain poorly understood [32].

Although T6SS expression was initially speculated to be energetically expensive for the cell, this assumption is now debated [4,33]. The complex regulatory networks controlling T6SS expression suggest a significant metabolic burden in vivo, potentially favoring heterogeneous expression to balance competitive advantage with growth [34]. However, recent studies in EAEC, *Vibrio cholerae*, *Vibrio fischeri*, and *Bacteroides fragilis* found that T6SS assembly is not energetically costly under laboratory conditions [35–39]. These studies, however, may not capture potential costs under more complex in vivo conditions. Indeed, T6SS expression in *Campylobacter jejuni* reduces fitness during bile salt exposure when competing with *E. coli* [38], and *Bacteroides fragilis* expressing T6SS incurs a fitness cost in the mouse gut but not in vitro [39].

Here, we investigate the molecular basis and ecological role of phenotypic heterogeneity of the *sci1* T6SS gene cluster of EAEC strain 17-2. Using fluorescent reporters, confocal microscopy, and microfluidics, we show that ON and OFF subpopulations coexist in equilibrium under iron-limiting conditions. ON or OFF subpopulations isolated revert to a heterogeneous state within approximately 10 generations, indicating epigenetic regulation. Mutational analysis of regulator-binding sites revealed that Fur binding at the distal F2 box and Dam methylation of the G3 site are key determinants of P*sci1* bistability. Finally, competition assays demonstrate that T6SS heterogeneity enhances population fitness in polymicrobial environments by enabling ON cells to attack competitors while OFF cells avoid retaliatory T6SS counterattacks. Our findings reveal phenotypic heterogeneity as a previously unrecognized strategy for fine-tuning T6SS activity and balancing competition with survival in polymicrobial communities.

## Results

### *sci1* expression is heterogeneous within clonal population of EAEC

**Fig 1:**
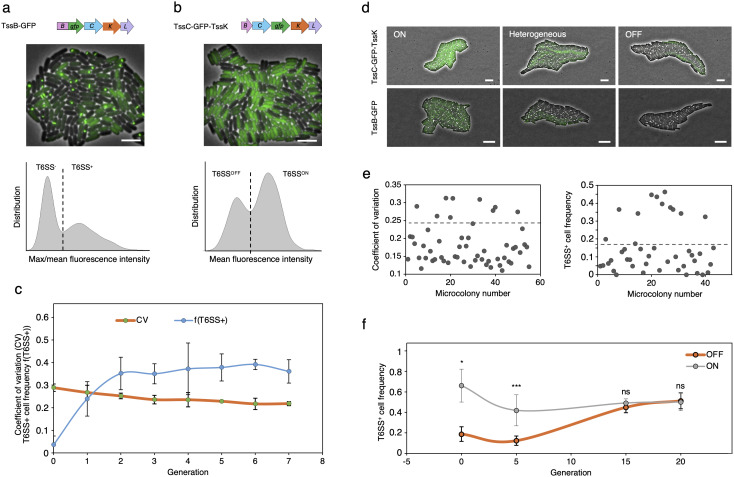
Phenotypic heterogeneity of T6SS expression and assembly reaches a stable equilibrium over generations. **(a)** Analyses of TssB-GFP cells. Representative confocal fields (top, merged bright and fluorescence channels; scale bar, 5 μm) and distribution of T6SS sheath (bottom, max/mean fluorescence intensity ratio) in TssB-GFP cells population. The y-axis represents the probability distribution of single-cell fluorescence intensities. **(b)** Analyses of TssC-GFP-TssK cells. Representative confocal fields (top, merged bright and fluorescence channels; scale bar, 5 μm) and distribution of fluorescence (bottom, mean fluorescence intensity ratio) in TssC-GFP-TssK cells population. The y-axis represents the probability distribution of single-cell fluorescence intensities. **(c)** Coefficient of variation (CV) of TssC-GFP-TssK populations and T6SS⁺ cell frequency (f(T6SS+)) in TssB-GFP populations as a function of generation number. T6SS⁺ cell frequency was calculated as the proportion of cells with a max/mean fluorescence ratio > 2. Approximately 2,000 cells were analyzed per generation from ≥3 independent replicates. The data represent means (circles) and standard deviations (SD, error bars) from ~2,000 cells per generation from ≥3 independent replicates. **(d)** Representative confocal fields of the 3 types of TssC-GFP-TssK (upper panels) or of TssB-GFP (lower panels) microcolonies (homogeneous ON, heterogeneous, homogeneous OFF). Scale bars, 10 μm. **(e)** Distribution of CV in TssC-GFP-TssK (left) and f(T6SS⁺) in TssB-GFP (right) across microcolonies from single-cell experiments. The dotted lines indicate the mean values. TssC-GFP-TssK: 8,500 cells analyzed (55 microcolonies from 4 independent replicates); TssB-GFP: 5,900 cells analyzed (43 microcolonies from 3 independent replicates). **(f)** Dynamics of f(T6SS^+)^ in sorted subpopulations over time. The data represent means (circles) ± standard deviations (SD, error bars) from at least three biological replicates. Statistical significance (one-tailed Wilcoxon’s *t t*est; ns, nonsignificant; *p < 0.1; ***p < 0.001) is indicated. The data underlying this Figure can be found in S1 Data.

Expression of the EAEC *sci1* T6SS gene cluster is repressed by Fur under iron-rich conditions, such as in LB medium, and induced under iron depletion, such as in M9/glycerol minimal medium (*sci1*-inducing medium, SIM [7]). Using a translational reporter strain expressing *tssB-sfGFP* to visualize T6SS sheath dynamics, we observed heterogeneous fluorescence in iron-limiting SIM medium (Fig 1A). Three distinct subpopulations were observed: cells that do not produce TssB-sfGFP (no fluorescence), cells that produce TssB-sfGFP (diffuse fluorescence) and cells that produce TssB-sfGFP and assemble sheaths (Figs 1A and S1). These observations suggest that heterogeneity occurs at both the transcriptional/translational level (expression/production versus no expression/production) and the post-translational level (assembly versus no assembly).

To gain further information on transcriptional heterogeneity, we engineered a reporter strain in which *sfGFP* was inserted between the *tssC* and *tssK* genes (TssC-GFP-TssK). This construct revealed a bimodal distribution of fluorescence (Fig 1B), with approximately 40% of cells in a low-fluorescence OFF state and 60% in a high-fluorescence ON state (Figs 1B and S1). The partial overlap between peaks (Fig 1B) suggests the presence of intermediate or transient activation states. Flow cytometric sorting of ON and OFF subpopulations confirmed the coexistence of these states (S4A Fig, see below).

### The ON/OFF equilibrium of *sci1* T6SS expression is stable across generations

To quantify heterogeneity, we calculated the coefficient of variation (CV) of single-cell fluorescence, i.e., the ratio between the standard deviation of the fluorescence represented by values from each cell with the mean fluorescence of the population (CV = SD/µ), over generations. With this parameter, higher CV values indicate greater heterogeneity. Both the CV and the proportion of T6SS⁺ cells remained stable over multiple generations in SIM, indicating that the ON/OFF equilibrium is maintained (Figs 1C and S2).

### Heterogeneous expression is stable and heritable across generations

To investigate how heterogeneity arises and propagates, we performed single-cell time-lapse microscopy using the TssB-GFP and TssC-GFP-TssK reporter strains. Cells grown to equilibrium were plated on a microfluidic chip at single-cell density. Individual founder cells gave rise to predominantly OFF, ON, or heterogeneous microcolonies after 7–10 generations (Figs 1D and S3). ON-enriched and heterogeneous colonies were more frequent than OFF-enriched colonies, resulting in a population distribution similar to that observed in liquid culture (CV > 0.20; Fig 1E). The overall frequency of T6SS⁺ cells was reduced in microfluidics, likely reflecting slower growth associated with lower oxygen availability compared with liquid cultures (Fig 1E). The presence of mostly homogeneous colonies indicates that the ON or OFF state can be transmitted from mother to progeny, with ON states appearing more frequently. It is, however, noteworthy that following transcriptional repression, visualization of the OFF state by fluorescence microscopy depends on the half-life of sfGFP.

To further assess the stability of the T6SS ON and OFF states, subpopulations were isolated by flow cytometry. The ratio of OFF and ON cells was comparable to that observed by fluorescence microscopy (S4A and S4B Fig). Sorted cells largely retained their activation state in the short term: ON cells predominantly formed ON colonies, and OFF cells predominantly formed OFF colonies (Figs 1F, S4C and S4D). However, both subpopulations reverted to the heterogeneous ON/OFF equilibrium within 15–20 generations in SIM (Fig 1F).

### Fur binding and GATC methylation govern heterogeneous P*sci1* expression

**Fig 2:**
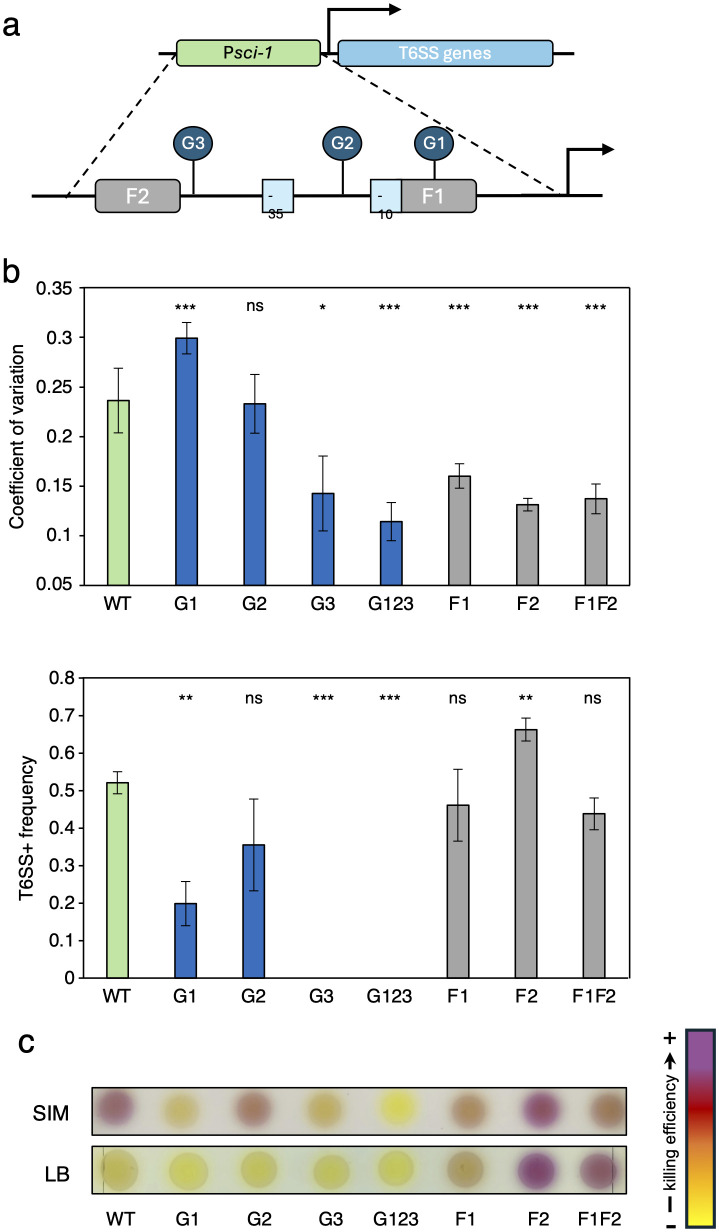
Fur and Dam binding sites F2 and G3 control heterogeneous T6SS expression. **(a)** Schematic representation of the *sci1* T6SS promoter (P*sci1*). P*sci1* contains two Fur binding boxes (F1 and F2) and three Dam methylation GATC sites (G1, G2, and G3). The F1 box includes the G1 site and overlaps with the transcriptional −10 element [7]. **(b)** TssB-GFP f(T6SS^+^) (bar plots) and TssC-GFP-TssK CV (line points) for WT and promoter variants. The data represent means (vertical bars) ± SD (error bars) from at least three biological replicates. Statistical significance compared to the WT strain (one-tailed Wilcoxon’s *t* test; ns, nonsignificant; *p < 0.1; **p < 0.01; ***p < 0.001) is indicated. **(c)** Competition assay between EAEC WT or promoter variants (attackers) and sensitive *E. coli* W3110 (recipient) in SIM (top) or LB (bottom) medium. Killing activity was visualized by the LAGA method [40], where color intensity reflects attacker activity (from yellow (no killing) to purple (intense killing), see heatmap on right). Images are representative results of biological triplicates. The data underlying this Figure can be found in S1 Data.

The ability of ON and OFF subpopulations to revert to equilibrium suggested that *sci1* heterogeneity is genetically controlled. The *sci1* promoter (P*sci1*) is comprised of multiple regulatory elements, including two Fur boxes (proximal F1 and distal F2) and three GATC sites, targeted by the Dam methyltransferase (proximal G1 to distal G3) (Fig 2A) [7]. G1 lies within F1, overlapping the −10 element, G2 is located between the −10 and −35 elements, while F2 and G3 are upstream of the −35 (Fig 2A). Fur binding onto F1 and F2 represses *sci1* expression and prevents G1 methylation, whereas G1 methylation antagonizes Fur binding, thereby promoting transcription [7].

To dissect the contributions of these regulatory elements to heterogeneity, we engineered chromosomal point mutations disrupting individual Fur boxes or GATC sites (S6A Fig). Preventing adenine methylation at GATC sites reduced P*sci1* activity, as shown by decreased T6SS sheath formation in G1, G3, and triple G123 mutants (Fig 2B). Conversely, disrupting the F2 Fur box relieved repression as the F2 mutant exhibited increased T6SS sheath numbers (Figs 2B and S5). When assessed in the TssC-GFP-TssK reporter strain, Fur box mutants displayed an increased ON state, whereas G3 and G123 mutants were homogeneously OFF (Figs 2B, S6B and S7). G1 and G2 mutants remained heterogeneous but with ON/OFF ratios distinct from wild-type (Figs 2B, S6 and S7). We thus concluded that the promoter architecture, via competing Fur binding and Dam methylation, is essential for establishing heterogeneous *sci1* expression.

We next assessed whether these mutations affected T6SS-mediated killing of *E. coli* W3110 target cells using the LAGA assay [40]. Figure 2C shows that the T6SS activity roughly correlated with the proportion of T6SS⁺ cells: G2 mutant cells exhibited reduced killing, while G1 and G3 cells were significantly impaired in killing and G123 mutant cells were inactive under inducing conditions (SIM, Fig 2C). In contrast, the F2 mutant showed increased T6SS activity compared to the WT and killed target cells even under iron-rich, repressive conditions (LB, Fig 2C). These findings support the model that ON cells deploy T6SS for antagonism, whereas OFF cells remain inactive.

### T6SS heterogeneity acts as an attenuation strategy against retaliatory T6SS attacks

Because the F2 and G123 mutants mimic homogeneous ON (T6SS^+^) and OFF (T6SS^−^) subpopulations, respectively, we used these strains to explore the functional significance of heterogeneity, particularly the role of OFF cells. One long-standing hypothesis is that T6SS imposes a substantial energetic burden, favoring heterogeneous expression as a cost-sharing strategy. However, consistent with previous findings [36], neither overexpression (F2) nor complete inhibition (G123) affected growth in liquid culture (S8A Fig) even in the presence of bile salts to mimic the EAEC ecological environment, indicating that T6SS assembly and activity are not energetically costly in EAEC and that the OFF subpopulation does not serve to counteract the growth defects associated to the production, assembly or activity of the T6SS.

As the T6SS was recently shown to serve as a foraging strategy to acquire nutrients by lysing neighboring cells [41–43], we next considered a suicide model in which OFF cells act as a nutritional reservoir for ON cells. In this case, OFF cells will not produce the Tli1 immunity protein and therefore can be killed by ON cells to support their growth. To test this hypothesis, we conducted competition experiments using the SGK assay [40]. The SGK assay is a growth recovery method that measures the time necessary for surviving bacteria to reach mid-exponential phase, which is inversely proportional to the initial number of surviving cells. We did not detect kin killing in EAEC: contrarily to Δ*sci1* mutant cells (i.e., deleted of the whole T6SS gene cluster) that are susceptible to WT attacks, G123 cells were not (S8B Fig). In addition, competition with the ON-homogeneous F2 strain did not increase mortality of WT or of G123 OFF cells (S8B Fig). This result suggests that basal expression of the immunity gene *tli1* from the internal promoter P_*4532*_ [44] is sufficient to confer protection against kin ON cells.

A third possibility is that T6SS heterogeneity is an attenuation strategy into the host. During infection, the immune system recognizes virulence factors to neutralize pathogens. An attenuation strategy, such as LPS modification in *Burkholderia pseudomallei* [45] or antigenic variation in *Borrelia burgdorferi* [46], allows to escape the immune system. However, we observed that homogeneous ON populations exhibited the highest infective success in *Galleria mellonella*, whereas OFF cells alone failed to efficiently colonize this wax moth host model (S8C Fig), arguing against this hypothesis.

**Fig 3:**
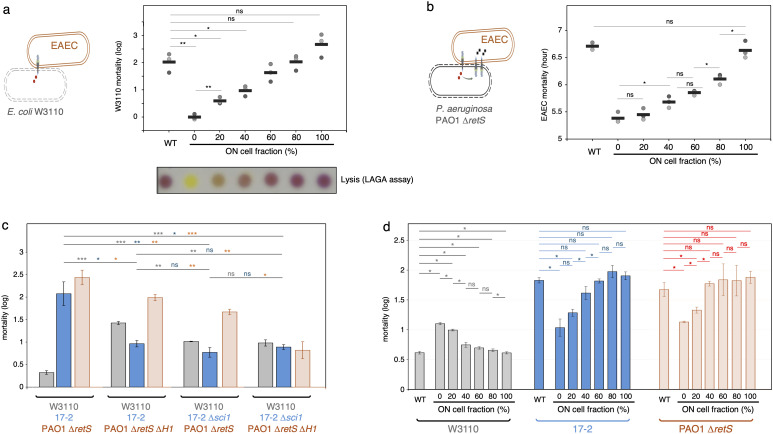
Phenotypic heterogeneity modulates T6SS strength and mediates a trade-off between killing efficiency and survival against T6SS⁺ competitors. **(a)** Competition assays between WT EAEC or mixed populations with increasing ON/OFF ratios (0%–100% ON) and *E. coli* W3110 recipients. F2 and G123 mutants were used as ON and OFF populations, respectively, and combined at different ratios to generate distinct heterogeneity states. Recipient mortality was quantified by the SGK method [40]. The data represent the three independent values (gray circles) and means (horizontal bars) from three biological replicates. Statistical significance between strains (Student *t* test; ns, nonsignificant; *p < 0.1; **p < 0.01) is indicated. **(b)** Competition assays between WT EAEC or ON/OFF mixtures and the T6SS⁺ defensive bacterium *Pseudomonas aeruginosa* PAO1Δ*retS*. EAEC mortality was quantified by the SGK method [40]. Data are means of 3 replicates. The data represent the three independent values (gray circles) and means (horizontal bars) from three biological replicates. Statistical significance between strains (Student *t* tes*t*; ns, nonsignificant; *p < 0.1) is indicated. **(c)** Three-species competition assays between *E. coli* K-12 W3110 (gray), EAEC strain 17-2 (WT or Δ*sci1*, blue), and *P. aeruginosa* PAO1Δ*retS* or PAO1Δ*retS*ΔH1-T6SS (orange) at a 1:1:1 ratio. Mortality of each bacterial species was quantified by the SGK method [40]. **(d)** Three-species competition assays between *E. coli* K-12 W3110 (gray), EAEC strain 17-2 (WT or increasing ON/OFF ratios (0%–100%), blue), and *P. aeruginosa* PAO1Δ*retS* or PAO1Δ*retS*ΔH1-T6SS (orange) at a 1:1:1 ratio. Mortality of each bacterial species was quantified by the SGK method [40]. The data represent means (vertical bars) ± SD (error bars) from nine replicates (technical triplicates of three biological replicates). Statistical significance compared to the WT strain (one-tailed Wilcoxon’s *t* test; ns, nonsignifican*t*; *p < 0.1; **p < 0.01; ***p < 0.001) is indicated with the strain color code. The data underlying this Figure can be found in S1 Data.

Finally, we hypothesized that heterogeneity mitigates antagonism in polymicrobial environments where defensive T6SS⁺ species deploy tit-for-tat retaliation [47]. Heterogeneous expression could thus serve as an attenuation strategy to reduce defence intensity and to improve survival. Consistent with this hypothesis, SGK assays showed that OFF cells (G123) alone failed to kill *E. coli* W3110, whereas increasing the ON/OFF ratio using synthetic mixes between the F2 and G123 populations proportionally enhanced antibacterial activity (Fig 3A). However, when competing with the model retaliatory T6SS-positive competitor *Pseudomonas aeruginosa* PAO1Δ*retS* (i.e., a strain that constitutively expresses the H1-T6SS but only assembles it and fires upon attacks from competitors [48]), increasing the ON/OFF ratio led to higher EAEC mortality (Fig 3B). These findings indicate that ON cells provide antagonistic capacity but simultaneously increase susceptibility to retaliatory T6SS attacks, whereas heterogeneity dampens this cost. Thus, T6SS heterogeneity functions as an attenuation strategy, balancing offensive capacity with survival.

To further evaluate the ecological relevance of EAEC T6SS *sci1* heterogeneity, we performed three-species competition assays with EAEC, the T6SS-negative and susceptible *E. coli* W3110 strain, and *P. aeruginosa* PAO1Δ*retS* mixed in a 1:1:1 ratio. Figure 3C shows that in this three-species competition assay, EAEC and *P. aeruginosa* engaged in reciprocal T6SS-mediated killing, leading to high mortality in both populations. Under these conditions, the strong reduction in EAEC abundance limited its ability to eliminate the susceptible W3110 strain. In contrast, when the H1-T6SS of *P. aeruginosa* was inactivated, EAEC efficiently eliminated both competitors while experiencing reduced mortality. Inactivation of both the EAEC Sci1 T6SS and *P. aeruginosa* H1-T6SS led to a stable equilibrium of the three strains during co-incubation.

To quantitatively assess the impact of phenotypic heterogeneity, we varied the proportion of T6SS-active (ON) cells within the EAEC population (Fig 3D). Increasing the fraction of ON cells enhanced killing of *P. aeruginosa*, but also resulted in an increase in EAEC mortality due to retaliatory attacks (Fig 3D). As a consequence, W3110 survival increased as EAEC populations became depleted through reciprocal killing.

## Discussion

In this work, we describe T6SS phenotypic heterogeneity and dissect the molecular and ecological basis of this behavior in EAEC. We show that in iron-limited inducing medium, the population stabilizes at a reversible equilibrium of ~60% ON and ~40% OFF cells. This equilibrium is not fixed: sorted ON or OFF subpopulations return to equilibrium within 15–20 generations, consistent with an epigenetic mechanism of control [8]. Similarly, growth in iron-rich medium yielded a homogeneous OFF population, yet dilution into minimal medium restored heterogeneity.

Our genetic analyses identify the P*sci1* promoter architecture, which comprises Fur boxes and GATC methylation sites [7], as the determinant of T6SS *sci1* heterogeneity. Mutations preventing Fur binding, notably F2 yield homogeneous ON populations, whereas mutations disrupting G3 methylation produce homogeneous OFF populations. These results align with the model in which Fur binding represses transcription and competes with Dam-mediated methylation at GATC sites [7]. In EAEC, Fur regulates the expression of the *sci1* T6SS gene cluster, while the methylation state of the GATC sites modulates Fur binding, allowing heterogeneous expression from cell to cell. Importantly, individual mutations at different promoter elements produce distinct ON/OFF ratios, suggesting that specific promoter architectures can tune the equilibrium between subpopulations. Such architectures may be subject to natural selection to optimize T6SS activity in specific ecological niches, consistent with recent modeling of promoter sequence variation and regulator affinity and processivity [49,50].

Our results confirm that Fur is the main regulator of the *sci1* gene cluster, with reversible binding to promoter Fur boxes controlling the ON/OFF switch [7]. Mutations of the Fur boxes, which prevent Fur binding, resulted in a constitutive ON state even in the presence of iron. T6SS heterogeneous expression, therefore, likely arises from the modulation of Fur binding. We ruled out heterogeneous *fur* transcription as the cause of T6SS heterogeneity, as P*fur* reporters are expressed uniformly in EAEC (S9 Fig). Instead, we propose that stochastic methylation of P*sci1* GATC sites modulates Fur binding, producing the observed distribution of expression states. Measuring the ON-OFF and OFF-ON switch rates in the different mutant backgrounds will likely provide further information to refine the regulatory model.

Interestingly, fluorescence microscopy and FACS analyses revealed a continuum of expression levels rather than two discrete peaks, suggesting intermediate states that may arise from hemi-methylated promoters or partial dilution of T6SS components during the ON-OFF switch. While our mutational analysis highlights key promoter elements (F2, G3), additional factors such as Dam and Fur abundance, iron availability, and methylation dynamics probably contribute to heterogeneity. Single-molecule tracking and microfluidics approaches [51] will be useful for elucidating these dynamics.

**Fig 4:**
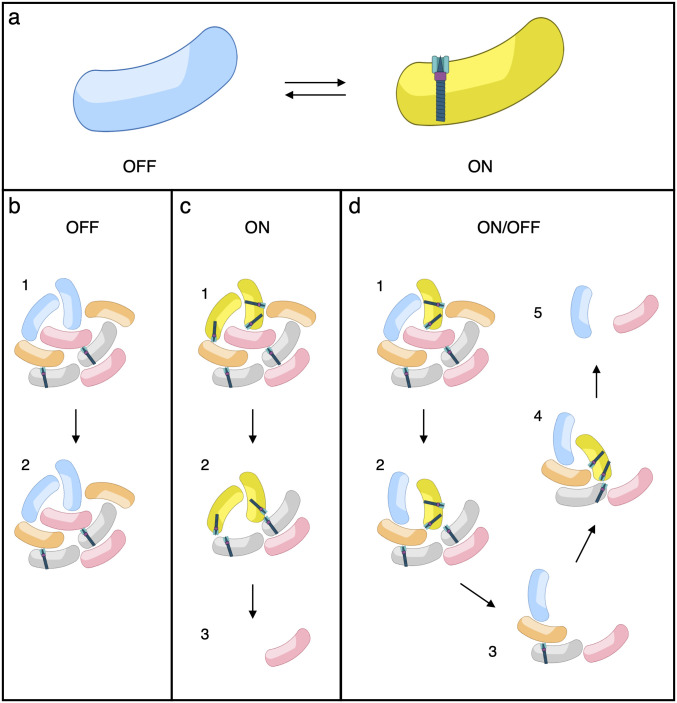
Model for the role of T6SS phenotypic heterogeneity in EAEC. **(a)** The *Psci1* promoter integrates Fur- and Dam-dependent regulation, generating ON (T6SS⁺, yellow) and OFF (T6SS⁻, blue) subpopulations at a reversible equilibrium. **(b)** OFF (blue) cells, which do not express T6SS, do not kill competitors but avoid triggering such defences (1). Due to the absence of killing activity, OFF cells are unable to colonize the niche (2). **(c)** While ON (yellow) cells assemble and deploy functional T6SS (1) and kill susceptible competitors (2), they provoke retaliatory attacks from defensive T6SS⁺ species (gray) and are eliminated and fail to colonize the niche (3). **(d)** Heterogeneous ON/OFF population (1) optimizes colonization by killing neighboring cells (2). While ON (yellow) cells are eliminated by counterattacks by defensive T6SS^+^ species (gray), OFF (blue) cells resist (3) and can ensure a continuous supply of ON cells (4-5) while limiting population-wide exposure to retaliation. Figure created with BioArt resources from NAID Visual & Medical Arts.

Functionally, ON cells confer antibacterial activity, whereas OFF cells do not contribute to killing but instead mitigate attacks from retaliatory T6SS⁺ competitors. Interestingly, our data suggest that killing efficiency scales approximately with the fraction of T6SS-active cells, whereas susceptibility to retaliatory killing displays a more nonlinear dependence, suggesting that costs can increase disproportionately at high ON fractions. EAEC T6SS heterogeneity, therefore, represents a trade-off that solves a conflict between two opposing pressures: the need for ON cells to eliminate susceptible recipients, and the cost of provoking counterattacks from armed competitors. Populations with a mixed ON/OFF composition thus maximize competitive advantage while avoiding excessive reciprocal killing (Fig 4). Recent work has shown that the competitive success of T6SS-armed bacteria depends not only on killing capacity per se, but also on activation dynamics, weapon deployment and associated costs. In *Vibrio fisheri*, faster activation of the T6SS can provide a competitive advantage, whereas excessive T6SS production can become counterproductive once its cost overweighs its benefits [52]. Our results further suggest that competition success may also depend on T6SS phenotypic heterogeneity operating as a frequency-dependent strategy that optimizes population fitness. This trade-off is particularly evident in multispecies contexts, where increasing the proportion of T6SS-active cells enhances killing but incurs a nonlinear survival cost. Such dynamics may represent an evolutionarily stable strategy in polymicrobial communities. Indeed, in communities such as the gut where hundreds of species cohabit, bacteria have developed antibacterial weapons but also defence mechanisms against these weapons. This includes capsule production, surface modification, cell aggregation [53–56]. However, these mechanisms carry substantial fitness costs [56,57]. The heterogeneity strategy described may enhance colonization success by combining offensive capacity with reduced susceptibility to retaliation. By modulating the fraction of ON cells, EAEC limits the activation of costly defensive strategies, representing an indirect form of adaptive defence. This ON/OFF balance may, however, come at a cost in other biological contexts. In particular, while OFF cells may be advantageous in interbacterial competition by limiting exposure to retaliatory attacks, they may be less effective in host-associated environments where T6SS activity contributes to virulence or niche establishment. Indeed, our results showed that ON cells better colonize the wax moth model, whereas OFF cells are less efficient. This context-dependent trade-off suggests that phenotypic heterogeneity may allow bacterial populations to balance competing selective pressures depending on the ecological conditions.‌‌

Our data support a model in which a homogeneous OFF population cannot invade due to a lack of killing, while a homogeneous ON population suffers from excessive counterattacks (Fig 4). In contrast, a heterogeneous population achieves maximal fitness by balancing competition and defence. Because ON/OFF states are reversible, OFF cells can regenerate the ON population lost during tit-for-tat killing, further supporting persistence in complex communities.

T6SS heterogeneity may extend beyond EAEC. Fluorescence microscopy recordings have revealed heterogeneous T6SS expression in many species, including *Burkholderia thailandensis*, *Pseudomonas putida*, *P. fluorescens*, *P. aeruginosa*, *Serratia marcescens*, *Salmonella enterica* Typhimurium*, Acinetobacter baylii*, *Y. pseudotuberculosis*, or *Vibrio fischeri* [16–22], despite their distinct regulatory circuits [5,6,21,22,58]. Indeed, in *Y. pseudotuberculosis*, *P. aeruginosa,* and EAEC, distinct regulatory architectures converge on similar heterogeneous outputs. Whereas T6SS heterogeneity in *Y. pseudotuberculosis* and *P. aeruginosa* depends on the RovC transcriptional regulator and c-di-GMP levels, respectively [21,22], our work demonstrates that EAEC T6SS heterogeneity arises from a complex interplay between Fur and DNA methylation regulations. Determining the molecular basis and functional significance of T6SS heterogeneity in other bacterial species will likely reveal the variety of regulatory mechanisms underlying heterogeneity and will clarify whether it represents a conserved trade-off strategy, a division-of-labor mechanism, or a bet-hedging strategy. Because T6SSs are employed to eliminate competitors in polymicrobial environment, we hypothesize that this attenuation strategy by a heterogeneous expression might be evolutionarily conserved.

In addition to heterogeneous gene expression, we observed heterogeneous T6SS sheath assembly using TssB-sfGFP fusions. Because subunit stoichiometry is known to be an important parameter controlling T6SS assembly, we propose that the heterogeneous assembly we observed could result from cell-to-cell differences in subunit abundance. Interestingly, a second promoter (P_*4532*_) within the cluster shares a similar architecture to P*sci1*, including overlapping Fur box, Dam site, and −10 element [44]. Future work should test whether this promoter also exhibits heterogeneity and whether it contributes to T6SS assembly by controlling expression of downstream T6SS genes. Another possibility is that accessory proteins involved in prey sensing and signaling [59–61] modulate T6SS activation at the post-translational level.

Given that gain and loss of T6SS loci are common across species [19,62–64], long-term fitness costs likely drive strong selection on T6SS regulation in vivo. EAEC appears to have evolved a finely tuned balance between T6SS activity and quiescence through phenotypic heterogeneity. This strategy may provide a general mechanism by which bacteria optimize the benefits of T6SS while limiting its costs in complex communities. Broader comparative analyses will further reveal whether this phenomenon is a common ecological strategy shaping bacterial interactions across diverse niches.

## Methods

### Bacterial strains and culture conditions

Bacteria and plasmids used in this study are listed in S1 Table. *E. coli* strains were routinely grown in LB medium (iron-rich conditions) or in *sci1* inducing minimal medium (SIM, M9 minimal medium supplemented with glycerol 0.2%, vitamin B1 200 μg/mL, casaminoacids 40 μg/mL, MgSO_4_ 1 mM and CaCl_2_ 0.1 mM; iron-starved conditions). When required, media were supplemented with antibiotics (chloramphenicol 40 µg/mL, kanamycin 50 µg/mL, ampicillin 100 µg/mL, gentamicin 15–20 µg/mL) or isopropyl-β-D-galactopyranoside (IPTG, 0.1 mM). Cultures were grown at 37 °C with shaking (180 rpm). Chloro-Phenol-Red-Galactopyranoside (CPRG, 2 mM) was prepared in distilled water.

### Plasmid construction

Plasmid and oligos used in this study are listed in S2 and S3 Tables, respectively. PCRs were performed using a Biometra thermocycler and Q5 polymerase (New England Biolabs). The pKO3-P*sci1* vector was generated by restriction-ligation. A 1-kb DNA fragment containing the P*sci1* promoter (from −500 pb to + 500 bp relative to the −10 element) was amplified from EAEC 17−2 genomic DNA with oligonucleotides carrying *Bam*HI and *Sal*I restriction sites at 5′ and 3′, respectively. The purified fragment and pKO3 plasmid were digested by BamHI and SalI (New England Biolabs) for 3 h at 37 °C and ligated with T4 DNA ligase (New England Biolabs) for 1 h at room temperature. Promoter point mutations were introduced by site-directed mutagenesis. Complementary oligonucleotide pairs bearing the desired substitution were used to amplify the entire pKO3-P*sci1* plasmid. PCR products were then digested with DpnI to remove template plasmids and transformed into DH5α competent cells. All pKO3-P*sci1* variants were verified by PCR and DNA sequencing (Eurofins Genomics).

### Strain construction

The fluorescence transcriptional reporter TssC-GFP-TssK was constructed by λ-Red recombination [65] using plasmid pKOBEG [66]. Electrocompetent cells harboring pKOBEG were transformed with a PCR product amplified from pKD4-Nt-sfGFP, which contained the sfGFP gene and a kanamycin cassette flanked by 50-bp extensions homologous to the upstream and downstream regions of the insertion site. Transformants were selected on kanamycin plates and verified by colony PCR before and sequencing of the site of insertion. The kanamycin cassette was then excised using pCP20 [65]. Directed mutagenesis was performed by allelic exchange using the pKO3 vector, which carries the *sacB* gene whose expression is toxic in the presence of sucrose [67]. Cells were transformed with pKO3-P*sci1* variants and incubated at 30 °C for plasmid replication. Clones were restreaked at 37 °C to select for chromosomal integration. Second crossover events were obtained by plating on LB agar plates supplemented with 5% sucrose at 37 °C. Colonies were restreaked on LB agar plates and randomly sequenced (Eurofins Genomics) to confirm the desired mutation.

### Competition assay

Intra-species competition assays were performed with EAEC 17-2 (or derivatives) as attacker strains and *E. coli* W3110 Kanᴿ or EAEC 17-2 (or derivatives) bearing a kanamycin-resistance cassette inserted into the *lacZ* gene [7] as recipients. Overnight cultures were diluted into fresh SIM medium and grown to an optical density at λ = 600 nm (OD_600_) of 0.8. For the lysis-associated β-galactosidase assay (LAGA), attacker and recipient strains were mixed at a 4:1 ratio; for the survivor growth kinetics assay (SGK), the ratio was 1:4 [40]. Mixtures (10 µL) were spotted on SIM agar and incubated for 4 h at 37 °C. In the qualitative LAGA assay, β-galactosidase released from lysed W3110 cells cleaves the membrane-impermeant β-galactosidase chromogenic substrate chlorophenol-red β-D-galactopyranoside (CPRG, yellow) into chlorophenol red (CPR, purple). 10 μL of CPRG 2 mM was then added on top of the spots. The color intensity correlates with killing efficiency. For quantitative SGK assays, spots were resuspended in LB supplemented with 50 μg/mL of kanamycin, serially 10-fold diluted in LB supplemented with kanamycin and inoculated in 96-well microplates. Recipient survivor growth was monitored using a TECAN microplate reader. Growth parameters were extracted with the R package growthcurver [68]. Because the time to resume growth depends on the initial cell concentration (i.e., the time required for the recipient population to resume exponential growth is inversely correlated with the initial number of surviving cells), the mid-exponential time point (Tmid) was used as a proxy for recipient mortality. For inter-species competition assays, *Pseudomonas aeruginosa* PAO1Δ*retS* was used as the attacker and EAEC wild-type strain 17-2 and its derivatives carrying the pBBR5-Gm^R^ vector as recipients. PAO1 was grown overnight in LB, diluted 25-fold, and cultured to OD₆₀₀ = 2. One mL was pelleted and resuspended to OD₆₀₀ = 10. EAEC strains were grown overnight in LB supplemented with 15 µg/mL gentamicin, diluted into SIM, and harvested at OD₆₀₀ = 0.8. Three-species competition assays were performed with EAEC 17-2 (or its Δ*sci1* derivative) with a kanamycin cassette inserted in the *lacZ* gene, PAO1 Δ*retS* (or its ΔH1-T6SS derivative) carrying the pBBR-Gm^R^ vector, and *E. coli* W3110 carrying the Amp^R^ pmCherry vector. Cells were grown as described above, mixed in a 1:1:1 ratio, and 10-μL mixtures were spotted on SIM agar plates. After 4h of incubation at 37 °C, spots were resuspended in LB and serially diluted in LB supplemented with 50 μg/mL of kanamycin, 20 μg/mL of gentamycin or 100 μg/mL of ampicillin for measuring the survival of EAEC, PAO1, and W3110, respectively. Mixtures containing defined F2(ON)/G123(OFF) ratios (0%–100%) were prepared and mixed with competitor cells, spotted on SIM agar, and incubated for 4 h at 37 °C and processed as above for quantitative measurements.

### Fluorescence microscopy

Cells were grown as described, concentrated 10-fold, and 2 µL were spotted onto a 2% agarose pad mounted in a Gene Frame. Slides were sealed with a coverslip and imaged on a Nikon Eclipse Ti2 microscope equipped with a 100 × NA 1.45 Ph3 objective, an ORCA-Fusiondigital camera (Hamamatsu) and a Perfect Focus System. sfGFP was excited at 488 nm. Images were analyzed with MicrobeJ after segmentation with MiSiC [69] to extract single-cell fluorescence. Distributions were plotted using ggplot2 and geom_density within the R package [70]. The coefficient of variation (CV = SD/mean) was used as a heterogeneity metric for TssC-GFP-TssK cell fluorescence data. Because the T6SS sheath gives the brighter pixel in a cell compared to a diffuse fluorescence, a ratio of maximum/mean pixel fluorescence > 2 was considered positive for sheath detection for strain TssB-GFP.

### Flow cytometry and cell sorting

Flow cytometry and fluorescence-activated cell sorting (FACS) were performed on an Influx v7 Cell Sorter (Becton) equipped with a stream-in-air 70 µm nozzle and a blue 488-nm Sapphire OPS laser (400 mW). The 488-nm laser was used for the analysis of the FSC (488/10 nm band pass filter, PMT1), which provides information related on cell size, and of the SSC (trigger signal, 488/10 nm band pass filter, PMT2), which provides information on cell density and the GFP fluorescence (530/40 nm band pass filter, PMT3). Light was detected with PMTs (Hamamatsu R3896 PMTs in C6270 sockets). FACSFlow buffer (Becton, Dickinson and Company) ran at 33 psi with an event rate of ~3,000 events s^−1^. The instrument was calibrated daily with 1-μm blue and 2-μm yellow-green FluoSphere beads (Thermo Fisher Scientific) in the linear range. For calibration in the logarithmic range and for spiking into the sample, 0.5-μm and 1-µm yellow-green beads (Thermo Fisher Scientific) were used. Acquisition was stopped after 50,000 events were recorded in the FSC against the SSC cell gate. FACS was performed as described [71] with the most accurate sorting mode ’1.0 drop pure’ and at a maximum sorting speed of 3,000 cells s^−1^. A total of 2 × 10^6^ cells per gate were collected for microscopy, mixed with glycerol (15% final concentration), and stored at −80 °C. 2D-plots were created using FlowJo V10 (Becton, Dickinson and Company). In addition, proportions of cells were analyzed in 2D-plots FSC versus green fluorescence in the GFP cell gate equal to 100% with respect to the 3 gates assembler, producer (ON) and nonproducer (OFF) for the 4 strains EAEC 17-2, TssB-GFP, TssC-GFP-TssK, and TssC-GFP-TssK F2, respectively. For the calculation, the beads in the GFP cell gate were subtracted.

### Single-cell experiments

Single-cell assays were performed in an Ibidi sticky-Slide VI 0.4. A 2% BD agar layer was poured into the channel, then cut to create a flow channel. The cell suspension was spotted on the agar, overlaid with a coverslip, and the input well was filled with 80 µL of culture medium. The device was incubated at 37 °C in a custom thermostable chamber on the microscope. Microcolonies growth was imaged after 4 h using the settings described above.

### Wax moth infection assay

*Galleria mellonella* larvae were used as an infection model. Larvae were starved for 48 h at 28 °C in the dark. EAEC 17-2 WT, F2 (ON) and G123 (OFF) strains bearing the kanamycin cassette inserted into the *lacZ* gene were grown to OD_600_ = 0.8. Starved larvae were infected by ingestion of 2 × 10^5^ CFU/mL and incubated for 24 h at 28 °C in the dark. After 24 h, larvae were washed for 10 s in sterile water, sacrificed, and their intern organs were collected and homogenized in 500 µL of PBS with 6 glass beads using a FastPrep-24 5G (MP biomedicals) for 30 s at power 4.5. Homogenates were serially diluted and plated on LB agar plate supplemented with 50 µg/mL of kanamycin for EAEC recovery. The assay was performed in triplicate.

### Statistical analysis

All statistical analyses were performed using R. Pairwise comparisons were conducted using unpaired two-tailed Student’s *t*-tests unless otherwise specified. For nonparametric datasets, statistical significance was assessed using one-tailed Wilcoxon tests. When comparing multiple groups, one-way ANOVA followed by Tukey’s post-hoc test was applied. All experiments were performed with at least three independent biological replicates.

## Supporting information

S1 Table Strains used in this study. (PDF)

S2 Table Plasmids used in this study. (PDF)

S3 Table Oligonucleotides used in this study. (PDF)

S1 Fig Phenotypic heterogeneity of T6SS expression and assembly. (PDF)

S2 Fig Phenotypic heterogeneity of T6SS expression and assembly over generations. (PDF)

S3 Fig Representative time-lapse recordings of a single-cell experiment from a clonal TssB-GFP cell. (PDF)

S4 Fig Cell sorting of subpopulations. (PDF)

S5 Fig T6SS sheath frequency in WT and F2 TssB-GFP cells. (PDF)

S6 Fig Impact of promoter point mutations on T6SS^+^ cell frequency (TssB-GFP) and T6SS expression (TssC-GFP-TssK). (PDF)

S7 Fig Phenotypic heterogeneity of the T6SS expression and assembly of promoter variants. (PDF)

S8 Fig Potential roles of T6SS heterogeneity. (PDF)

S9 Fig P*sci1* and P*fur* activity in EAEC. (PDF)

S1 Data Raw data. (XLSX)

## Abbreviations

CPRG: chloro-phenol-red-galactopyranoside
CV: coefficient of variation
EAEC: *Escherichia coli*
FACS: fluorescence-activated cell sorting
LAGA: lysis-associated β-galactosidase assay
P*sci1*: *sci1* promoter
SD: standard deviations
SGK: growth kinetics assay
T3SS: type III secretion system
T6SS: type VI secretion system
Tmid: mid-exponential time point.
