# Supplementary material for: Phenotypic heterogeneity optimizes trade-offs during adaptive deployment of the type VI secretion system
Source: PLoS Biol. 2026 Jun 4;24(6):e3003838. doi: 10.1371/journal.pbio.3003838 (PMC13262931; doi:10.1371/journal.pbio.3003838)
Supplement: S1 Table — (PDF) [file pbio.3003838.s001.pdf]

## S1 Table. Strains used in this study

| Strain | Description | Source |
|---|---|---|
| ***Escherichia coli* K-12** | | |
| DH5a | Genotype | New England Biolabs |
| W3110 | Genotype | K. Postle |
| **Enteroaggregative *Escherichia coli*** | | |
| 17-2 | | A. Darfeuille-Michaud |
| 17-2 Δ*sci1* | | Aschtgen et al., 2008 |
| 17-2 *lacZ*::kan | 17-2 with $Kan^R$ cassette inserted at *lacZ* locus | This study |
| 17-2 Δ*sci1* *lacZ*::kan | 17-2 Δ*sci1* with $Kan^R$ cassette inserted at *lacZ* locus | This study |
| 17-2 TssB-GFP | sfGFP fused to *tssB* | Zoued *et al.,*2013 |
| 17-2 TssC-GFP-TssK | sfGFP inserted between the *tssC* and *tssK* genes | This study |
| 17-2 F1 | P*sci1* Fur box1 mutant in 17-2 | This study |
| 17-2 F2 | P*sci1* Fur box2 mutant in 17-2 | This study |
| 17-2 F1F2 | P*sci1* Fur boxes mutants in 17-2 | This study |
| 17-2 G1 | P*sci1* GATC1->GGTC in 17-2 | This study |
| 17-2 G2 | P*sci1* GATC2->GGTC in 17-2 | This study |
| 17-2 G3 | P*sci1* GATC3->GGTC in 17-2 | This study |
| 17-2 G12 | GATC1/GATC2 double mutant in 17-2 | This study |
| 17-2 G123 | GATC/GATC2 /GATC3 triple mutant in 17-2 | This study |
| 17-2 TssB-GFP F1 | P*sci1* Fur box1 mutant in 17-2 TssB-GFP | This study |
| 17-2 TssB-GFP F2 | P*sci1* Fur box2 mutant in 17-2 TssB-GFP | This study |
| 17-2 TssB-GFP F1F2 | P*sci1* Fur boxes mutants in 17-2 TssB-GFP | This study |
| 17-2 TssB-GFP G1 | P*sci1* GATC1->GGTC in 17-2 TssB-GFP | This study |
| 17-2 TssB-GFP G2 | P*sci1* GATC2->GGTC in 17-2 TssB-GFP | This study |
| 17-2 TssB-GFP G3 | P*sci1* GATC3->GGTC in 17-2 TssB-GFP | This study |
| 17-2 TssB-GFP G12 | GATC1/GATC2 double mutant in 17-2 TssB-GFP | This study |
| 17-2 TssB-GFP G123 | GATC/GATC2 /GATC3 triple mutant in 17-2 TssB-GFP | This study |

| | | |
|---|---|---|
| 17-2 TssC-GFP-TssK F1 | P*sci1* Fur box1 mutant in 17-2 TssC-GFP-TssK | This study |
| 17-2 TssC-GFP-TssK F2 | P*sci1* Fur box2 mutant in 17-2 TssC-GFP-TssK | This study |
| 17-2 TssC-GFP-TssK F1F2 | P*sci1* Fur boxes mutants in 17-2 TssC-GFP-TssK | This study |
| 17-2 TssC-GFP-TssK G1 | P*sci1* GATC1->GGTC in 17-2 TssC-GFP-TssK | This study |
| 17-2 TssC-GFP-TssK G2 | P*sci1* GATC2->GGTC in 17-2 TssC-GFP-TssK | This study |
| 17-2 TssC-GFP-TssK G3 | P*sci1* GATC3->GGTC in 17-2 TssC-GFP-TssK | This study |
| 17-2 TssC-GFP-TssK G12 | GATC1/GATC2 double mutant in 17-2 TssC-GFP-TssK | This study |
| 17-2 TssC-GFP-TssK G123 | GATC/GATC2 /GATC3 triple mutant in 17-2 TssC-GFP-TssK | This study |
| ***Pseudomonas aeruginosa*** | | |
| PAO1 | Wild-type strain | |
| PAO1 Δ*retS* | Deletion of *retS* regulator in PAO1 | C. Kreuzer |
| PAO1 Δ*retS* ΔH1-T6SS | Deletion of *H1-T6SS gene cluster* in PAO1 Δ*retS* | C. Kreuzer |
