# Supplementary material for: Phenotypic heterogeneity optimizes trade-offs during adaptive deployment of the type VI secretion system
Source: PLoS Biol. 2026 Jun 4;24(6):e3003838. doi: 10.1371/journal.pbio.3003838 (PMC13262931; doi:10.1371/journal.pbio.3003838)
Supplement: S2 Table — (PDF) [file pbio.3003838.s002.pdf]

S2 Table. Plasmids used in this study

Plasmid	Features	Description
pJET	Amp^R	Cloning vector for quick change mutagenesis
pKO3	SacB, Cm^R, *ori*^{ts}	Suicide vector for chromosomal site-directed mutagenesis
pKO3-P*sci1*	P*sci1* DNA fragment cloned into pKO3	Suicide vector with DNA fragment encompassing P*sci1* promoter
pKO3-P*sci1*-F1	F1 mutation introduced into pKO3-P*sci1*	pKO3-P*sci1* with F1 mutation
pKO3-P*sci1*-F2	F2 mutation introduced into pKO3-P*sci1*	pKO3-P*sci1* with F2 mutation
pKO3-P*sci1*-F12	F2 mutation introduced into pKO3-P*sci1*-F1	pKO3-P*sci1* with F1 and F2 mutations
pKO3-P*sci1*-G1	G1 mutation introduced into pKO3-P*sci1*	pKO3-P*sci1* with G1 mutation
pKO3-P*sci1*-G2	G2 mutation introduced into pKO3-P*sci1*	pKO3-P*sci1* with G2 mutation
pKO3-P*sci1*-G3	G3 mutation introduced into pKO3-P*sci1*	pKO3-P*sci1* with G3 mutation
pKO3-P*sci1*-G12	G1 and G2 mutations introduced into pKO3-P*sci1*	pKO3-P*sci1* with G1 and G2 mutations
pKO3-P*sci1*-G123	G3 mutation introduced into pKO3-P*sci1*-G12	pKO3-P*sci1* with G1, G2 and G3 mutations
pKD4	FRT-Kan^R-FRT	Template for lambda-red recombination
pKOBEG	Rec genes, Cm^R, *ori*^{ts}	Plasmid-encoding lambda-red system
pBAD33	P*lac*, Cm^R	Expression vector
pPROBE-gfp-AAV	gfp-AAV, Kan^R	Transcriptional reporter plasmid with unstable GFP
pPROBE-gfp-AAV-Fur	P*fur*, gfp-AAV, Kan^R	Unstable GFP under the control of P*fur* promoter
pPROBE-gfp-AAV-Sci1	P*sci1*, gfp-AAV, Kan^R	Unstable GFP under the control of P*sci1* promoter
pBBR-MCS5	Gm^R	Broad Host Range plasmid with gentamycin gene
