# Supplementary material for: Phenotypic heterogeneity optimizes trade-offs during adaptive deployment of the type VI secretion system
Source: PLoS Biol. 2026 Jun 4;24(6):e3003838. doi: 10.1371/journal.pbio.3003838 (PMC13262931; doi:10.1371/journal.pbio.3003838)
Supplement: S3 Table — (PDF) [file pbio.3003838.s003.pdf]

S3 Table. Oligonucleotides used in this study

**Cloning into pJET/pKO3**	
5-pKO3-Psci-1	TATCGGATCCGCCGGCAGGGATTTCG
3-pKO3-Psci-1	ACGGTCGACCGGCATGCGCTTCAG
**Quick-change mutagenesis**	
5-G1	CAGATCTTCGTCCTATAATGGTCAAAATTAAATCAGTGCACAAG
3-G1	CTTGTGCACTGATTTAATTTTGACCATTATAGGACGAAGATCTG
5-G2	CTGTTATATTGAGATTTTTCAGGTCTTCGTCCTATAATG
3-G2	CATTATAGGACGAAGACCTGAAAAATCTCAATATAACAG
5-G1-2	CTGTTATATTGAGATTTTTCAGGTCTTCGTCCTATAATGGTCAAAATTA AATCAGTGCACAAGGG
3-G1-2	CCCTTGTGCACTGATTTAATTTTGACCATTATAGGACGAAGACCTGAA AAATCTCAATATAACAG
5-G3	CTGATTATTTGCATTATATCGGTCGATGTATCTGTTATATTG
3-G3	CAATATAACAGATACATCGACCGATATAATGCAAATAATCAG
5-F1	CGTCCTATAATGATCAAAAGGGAATCAGTGCACAAGGGGAGGC
3-F1	GCCTCCCCTTGTGCACTGATTCCCTTTTGATCATTATAGGACG
5-F2	TAAAGTCCTGATTATTTGCAGGGTATCGATCGATGTATCTGTT
3-F2	AACAGATACATCGATCGATACCCTGCAAATAATCAGGACTTTA
**Cloning into pPROBE**	
5-pPROBE-P*sci*1-SalI	TATCGTCGACTCATTGCATTTGTTGGGCCCT
3-pPROBE-P*sci1*-EcoRI	ACGGAATTCGGCTCTCTCCTGTGAAACCTGC
5-pPROBE-P*fur*-EcoRI	TATCGTCGACTCTCGGTCTGGCTATCGACG
3-pPROBE-P*fur*-SalI	ACGGAATTCGCGGAATCTGTCCTG
pKD4-Nt-sfGFP	
5-tssC-sfGFP-tssK	TGGACGTCAGCCTGTCACTGGTTTCGCAGATGCCGAAGGCAAAAGCG TAACGATTGTGTAGGCTGGAGCTGCTTCGAAGTTCCTATAC
3-tssC-sfGFP-tssK	GCCCCGTCTTCCCATAATGGGCGATAAATCTTCATTTCCCGACACCTG CCTTACCCTCCGCCGGCCGCTGC
