# Supplementary material for: Phenotypic heterogeneity optimizes trade-offs during adaptive deployment of the type VI secretion system
Source: PLoS Biol. 2026 Jun 4;24(6):e3003838. doi: 10.1371/journal.pbio.3003838 (PMC13262931; doi:10.1371/journal.pbio.3003838)
Supplement: S1 Fig — (PDF) [file pbio.3003838.s004.pdf]

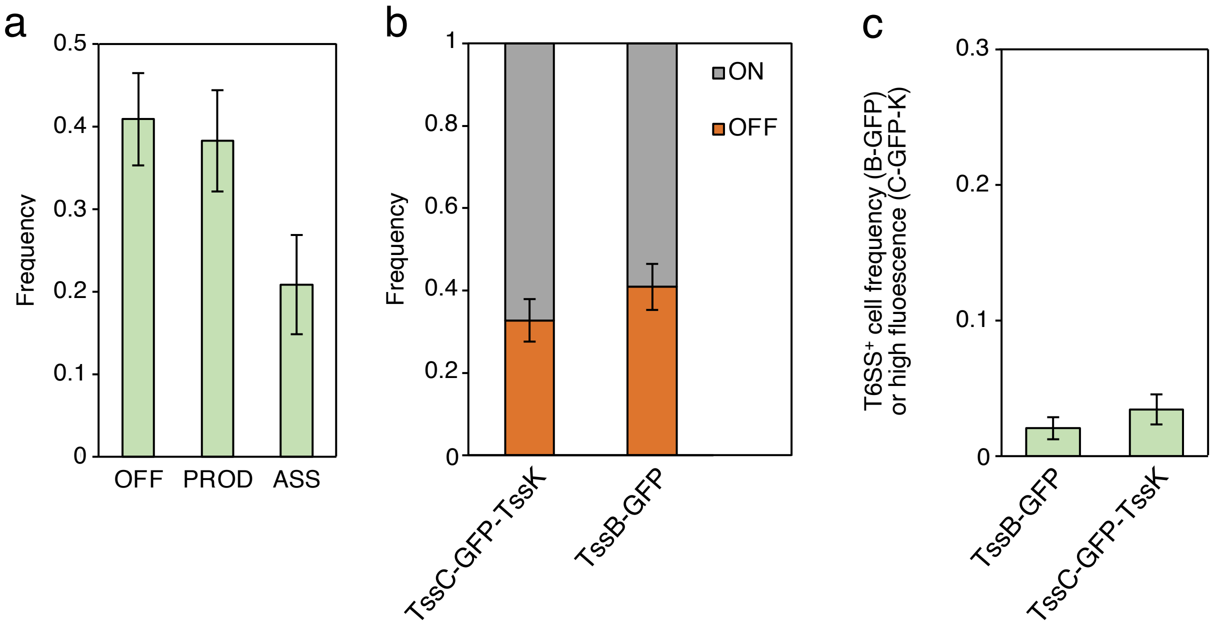

S1 Figure | Phenotypic heterogeneity of T6SS expression and assembly. (a) Frequency of cell with no sheath (OFF), TssB production (PROD) or assembled T6SS (ASS) in a clonal population of EAEC TssB-GFP. The data represent means (vertical bars) ± SD (error bars) from 5,450 cells from 3 independent replicates. **(b)** Frequency of ON and OFF cells in clonal TssC-GFP-TssK or TssB-GFP EAEC population. The data represent means (vertical bars) ± SD (error bars) from 11,760 (TssC-GFP-TssK) and 5,450 (TssB-GFP) cells from 3 independent replicates. **(c)** T6SS^{+} cell frequency (TssB-GFP) or fluorescence (TssC-GFP-TssK) in iron-rich LB medium. The data represent means (vertical bars) ± SD (error bars) from 1,945 (TssC-GFP-TssK) and 1,518 (TssB-GFP) cells from 3 independent replicates. The data underlying this Figure can be found in S1 Data.
