# Supplementary material for: Phenotypic heterogeneity optimizes trade-offs during adaptive deployment of the type VI secretion system
Source: PLoS Biol. 2026 Jun 4;24(6):e3003838. doi: 10.1371/journal.pbio.3003838 (PMC13262931; doi:10.1371/journal.pbio.3003838)
Supplement: S2 Fig — (PDF) [file pbio.3003838.s005.pdf]

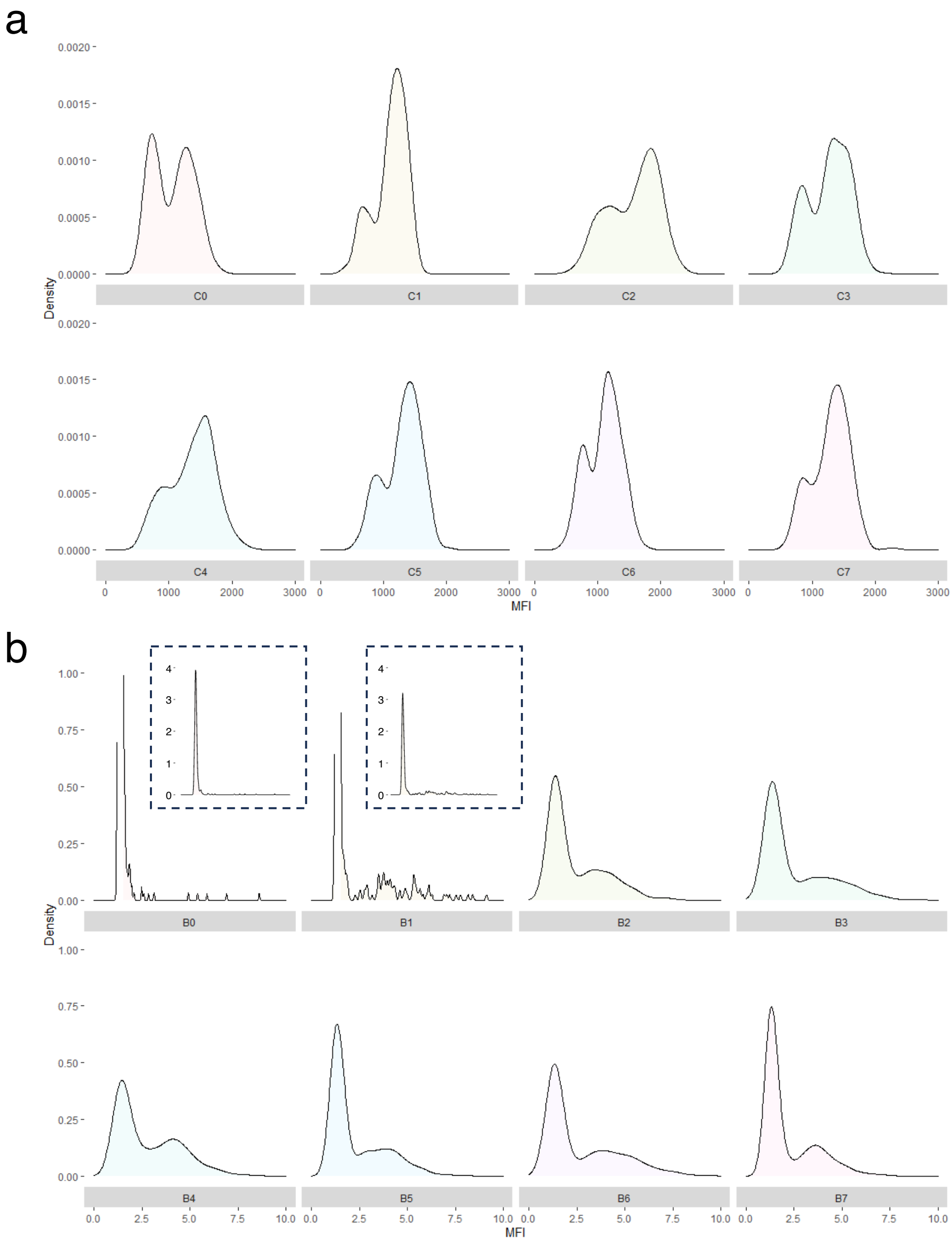

S2 Figure | Phenotypic heterogeneity of T6SS expression and assembly over generations. (a) Representative distribution (Density) of the mean fluorescence intensity (MFI) of TssC-GFP-TssK cells in the populations over seven generations (C0 to C7) (*n*>400 cells for each generation). **(b)** Representative distribution of max/mean fluorescence intensity ratio (sheath detection) of TssB-GFP cells in the population over seven generations (B0 to B7) (*n*>300 cells for each generation). For clarity, the *y*-axis scale of the B0 and B1 plots was adjusted to facilitate comparison between distributions with different peak amplitudes. As a result, the highest peaks are partially truncated in the main panels. Full distributions are shown in the inset panels.
