# Supplementary material for: Phenotypic heterogeneity optimizes trade-offs during adaptive deployment of the type VI secretion system
Source: PLoS Biol. 2026 Jun 4;24(6):e3003838. doi: 10.1371/journal.pbio.3003838 (PMC13262931; doi:10.1371/journal.pbio.3003838)
Supplement: S3 Fig — (PDF) [file pbio.3003838.s006.pdf]

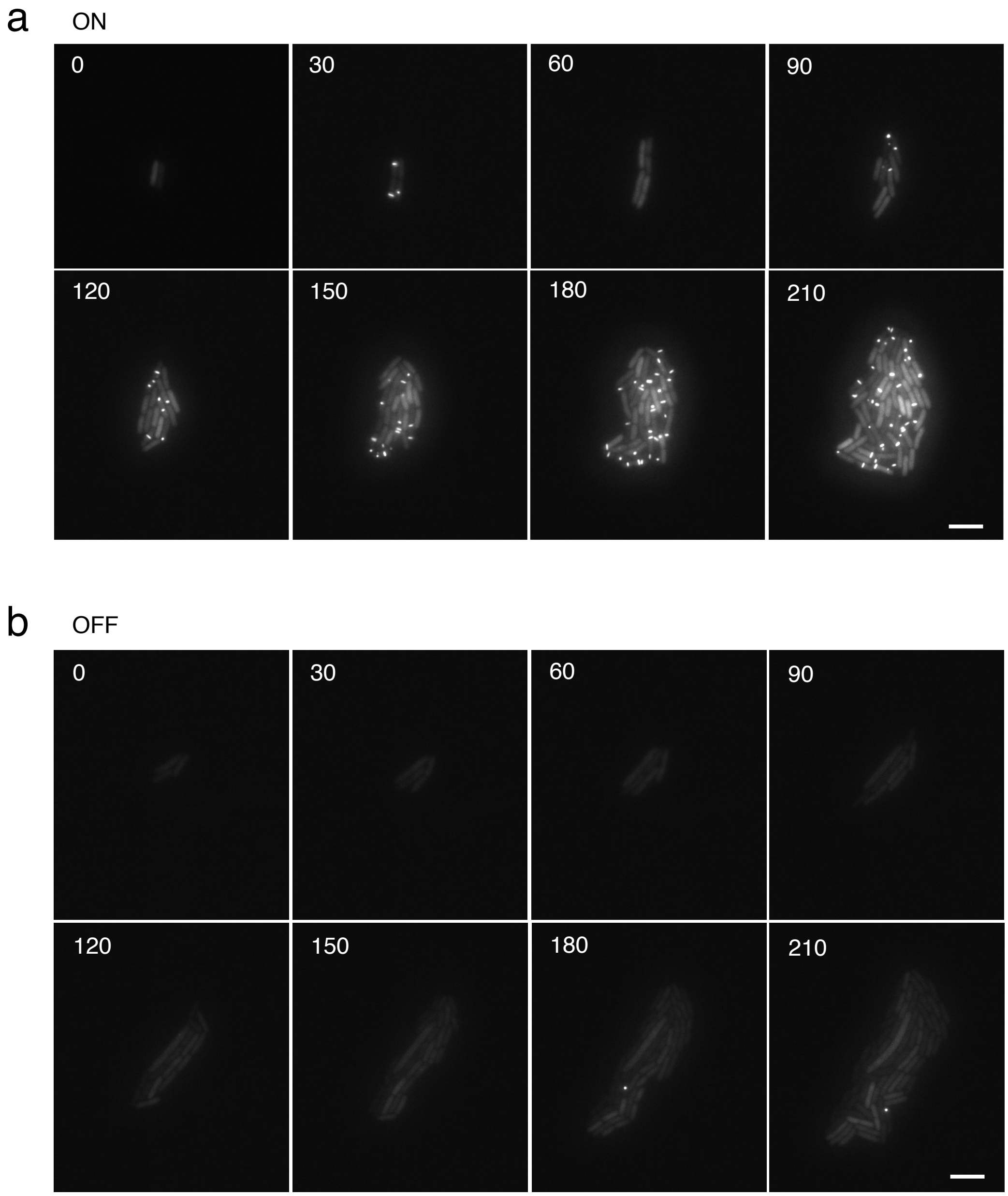

S3 Figure | Representative time-lapse recordings of single-cell experiment from a clonal TssB-GFP cell. Single-cell imaging of a ON (a) and OFF (b) cell grown in a microfluidic device fed with SIM medium. Images were taken every 30 minutes (time, in minutes, indicated on each panel). Scale bars, 10 µm.
