# Supplementary material for: Phenotypic heterogeneity optimizes trade-offs during adaptive deployment of the type VI secretion system
Source: PLoS Biol. 2026 Jun 4;24(6):e3003838. doi: 10.1371/journal.pbio.3003838 (PMC13262931; doi:10.1371/journal.pbio.3003838)
Supplement: S4 Fig — (PDF) [file pbio.3003838.s007.pdf]

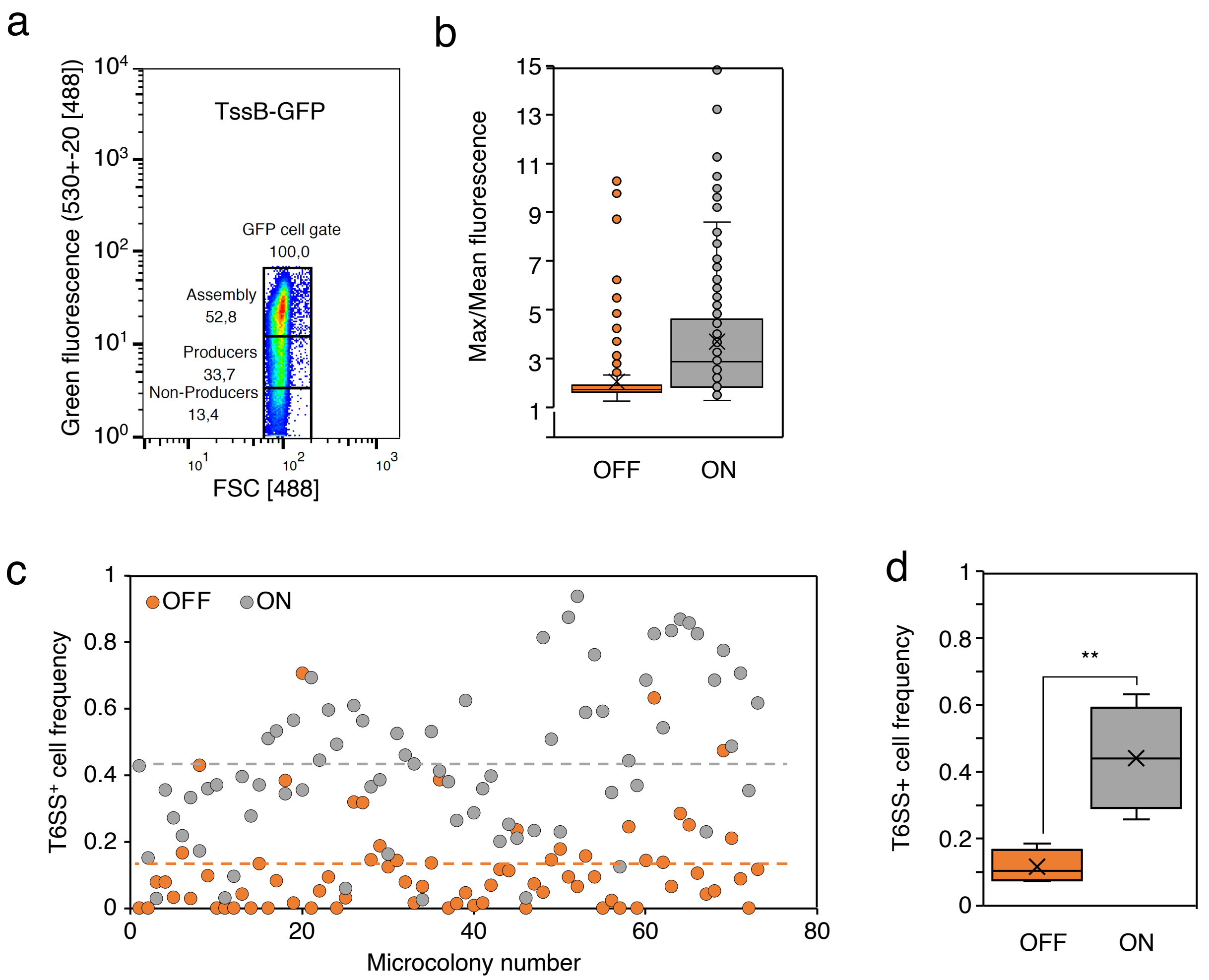


**S4 Figure | Cell sorting of subpopulations. (a)** Distribution of the mean fluorescence of a TssB-GFP population determined by flow cytometry at 6 h of culture after 1/100 dilution of a preculture in SIM+LB 10%. Non producers gate was defined with the autofluorescence of EAEC 17-2 strain; Assembly gate was defined with the fluorescence distribution of TssC-GFP-TssK F2; Producers gate was defined as the aera of medium intensity between Non-producers and Assembly gates. **(b)** Box plot representation of the quality control of the cell sorting of ON (assembler and producer) and OFF (non-producer) subpopulations. The presence of a sheath is determined by a max and mean fluorescence ratio >2. The horizontal bar represents the median value; the cross represents the mean; the lower and upper boundaries of the box plot correspond to the 25th and 75th percentiles respectively, whereas the whiskers represent the SD. Each circle represents a cell ($n$=175 and 184 OFF and ON cells analysed from 2 independent samples, respectively). **(c)** T6SS$^+$ cell frequency in microcolonies from single-cell experiments of sorted ON and OFF subpopulations. Each dot is a microcolony. The overall average frequency is shown as a dotted line with the corresponding colour. More than 3,400 cells were analysed from 73 microcolonies from 6 independent replicates. The statistical analyses (Box plot representation with median values (horizontal bars), mean (crosses), 25$^{th}$ and 75$^{th}$ percentiles (lower and upper boundaries) ± SD (error bars) from $n$=6 cells) is shown in panel **(d)**. Statistical significance compared between the two populations (one-tailed Wilcoxon's $t$-test; **, $p$<0.01) is indicated. The data underlying this Figure can be found in S1 Data.
