# Supplementary material for: Phenotypic heterogeneity optimizes trade-offs during adaptive deployment of the type VI secretion system
Source: PLoS Biol. 2026 Jun 4;24(6):e3003838. doi: 10.1371/journal.pbio.3003838 (PMC13262931; doi:10.1371/journal.pbio.3003838)
Supplement: S5 Fig — (PDF) [file pbio.3003838.s008.pdf]

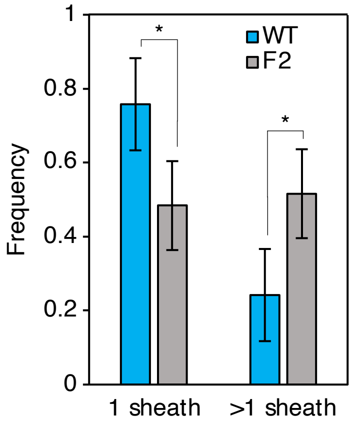

S5 Figure | T6SS sheath frequency in WT and F2 TssB-GFP cells. Statistical analyses reporting the percentage of WT (blue bars) and F2 (grey bars) cells assembling one or more sheaths per cell. Only cells with dynamic sheaths were analyzed. The data represent means (vertical bars) ± SD (error bars) from 168 (WT) and 185 (F2) cells, from 8 fields from 4 biological replicates (2 fields per replicate). Statistical significance between the WT and F2 strains (one-tailed Wilcoxon's *t*-test; *, $p < 0.1$) is indicated. The data underlying this Figure can be found in S1 Data.
