# Supplementary material for: Phenotypic heterogeneity optimizes trade-offs during adaptive deployment of the type VI secretion system
Source: PLoS Biol. 2026 Jun 4;24(6):e3003838. doi: 10.1371/journal.pbio.3003838 (PMC13262931; doi:10.1371/journal.pbio.3003838)
Supplement: S6 Fig — (PDF) [file pbio.3003838.s009.pdf]

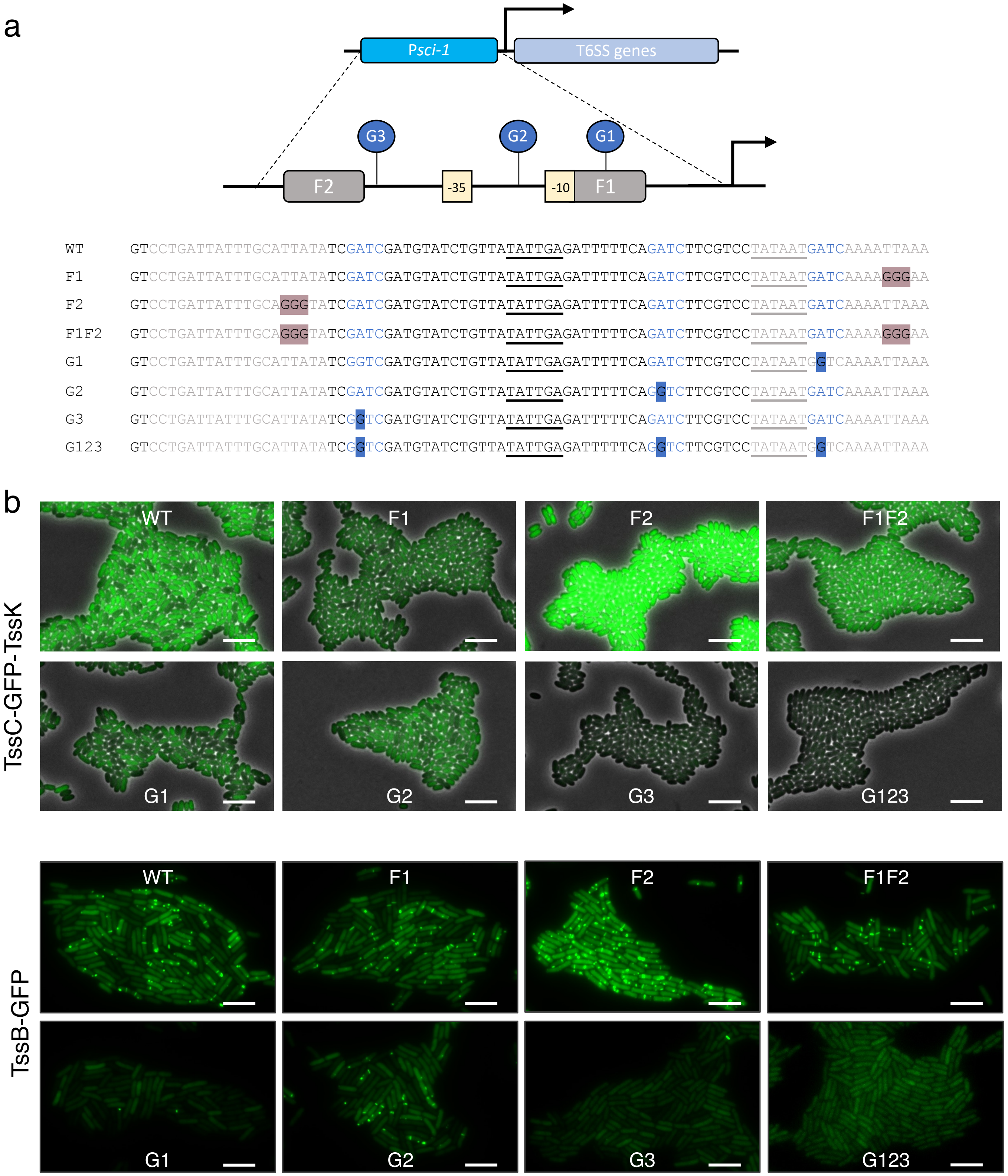

S6 Figure | Impact of promoter point mutations on T6SS^{+} cell frequency (TssB-GFP) and T6SS expression (TssC-GFP-TssK). (a) Sequence alignment of WT and variant P*sci1*. Fur boxes sequences are coloured in grey, GATC sites in blue and -10 and -35 elements are underlined. Fur boxes were mutated by the substitution of 3 bases (GGG highlighted in grey) to break the palindrome. GATC sites were mutated by the substitution of the Adenine by a Guanosine (G highlighted in blue) to prevent methylation. **(b)** Representative fields of TssC-GFP-TssK cells in different genetic backgrounds. Images are merged channels of phase and fluorescence set up with the same minimal and maximal signal scale. TssB-GFP images are only fluorescence images to better observe T6SS sheaths. G3 and G123 are overexposed to distinguish cells. Scale bars, 10 µm.
