# Supplementary material for: Phenotypic heterogeneity optimizes trade-offs during adaptive deployment of the type VI secretion system
Source: PLoS Biol. 2026 Jun 4;24(6):e3003838. doi: 10.1371/journal.pbio.3003838 (PMC13262931; doi:10.1371/journal.pbio.3003838)
Supplement: S7 Fig — (PDF) [file pbio.3003838.s010.pdf]

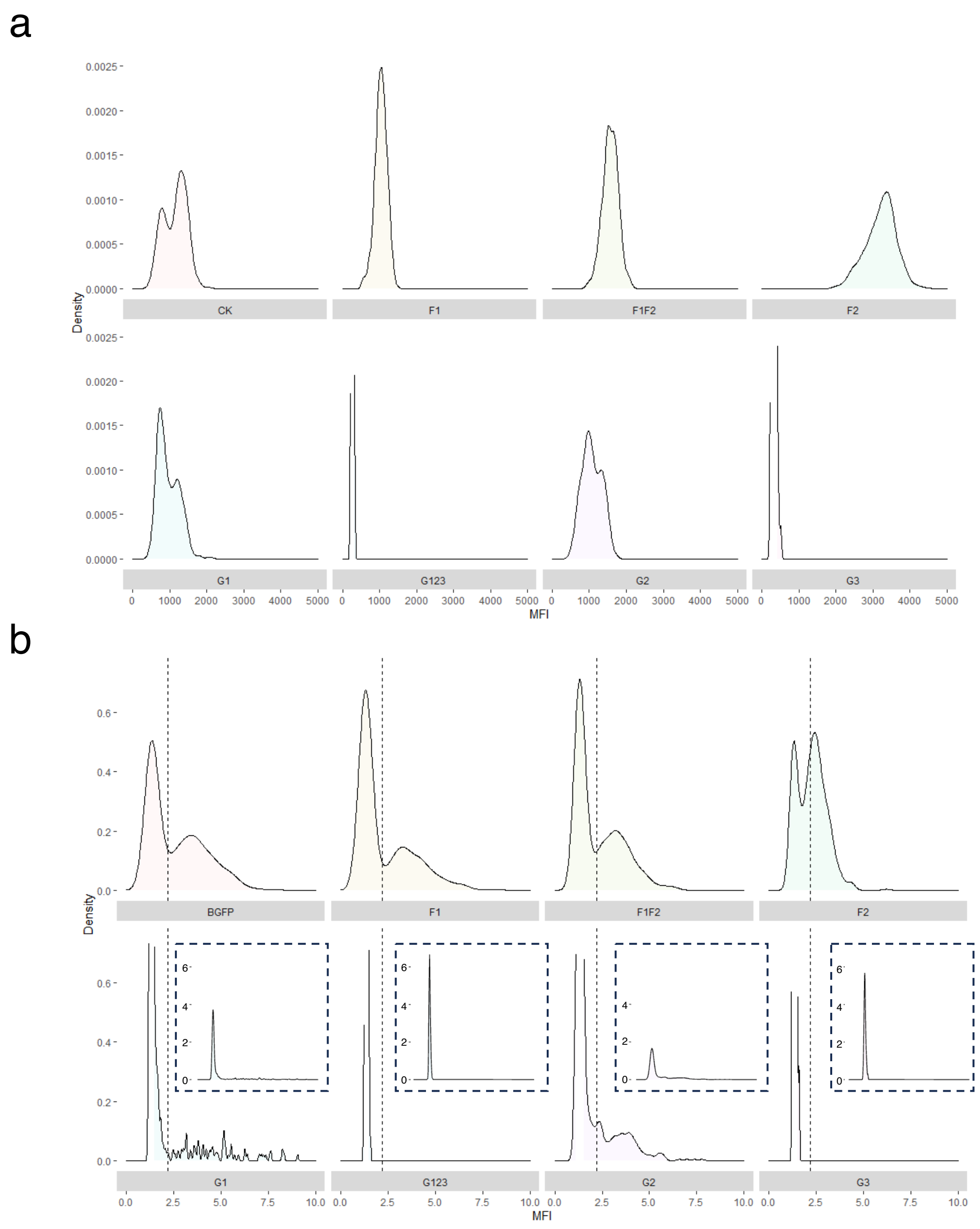

S7 Figure | Phenotypic heterogeneity of the T6SS expression and assembly of promoter variants. (a) Representative distribution (Density) of the mean fluorescence (MFI) of TssC-GFP-TssK populations in promoter variants. >500 cells per strains were analysed, from 3 independent replicates. **(b)** Representative distribution of max/mean fluorescence ratio (sheath detection) of TssB-GFP populations in promoter variants. >500 cells per strains were analysed, from 3 independent replicates. For clarity, the *y*-axis scale of the main G1, G2, G3 and G123 plots was adjusted to facilitate comparison between distributions with different peak amplitudes. As a result, the highest peaks are partially truncated in the main panels. Full distributions are shown in the inset panels.
