# Supplementary material for: Phenotypic heterogeneity optimizes trade-offs during adaptive deployment of the type VI secretion system
Source: PLoS Biol. 2026 Jun 4;24(6):e3003838. doi: 10.1371/journal.pbio.3003838 (PMC13262931; doi:10.1371/journal.pbio.3003838)
Supplement: S8 Fig — (PDF) [file pbio.3003838.s011.pdf]

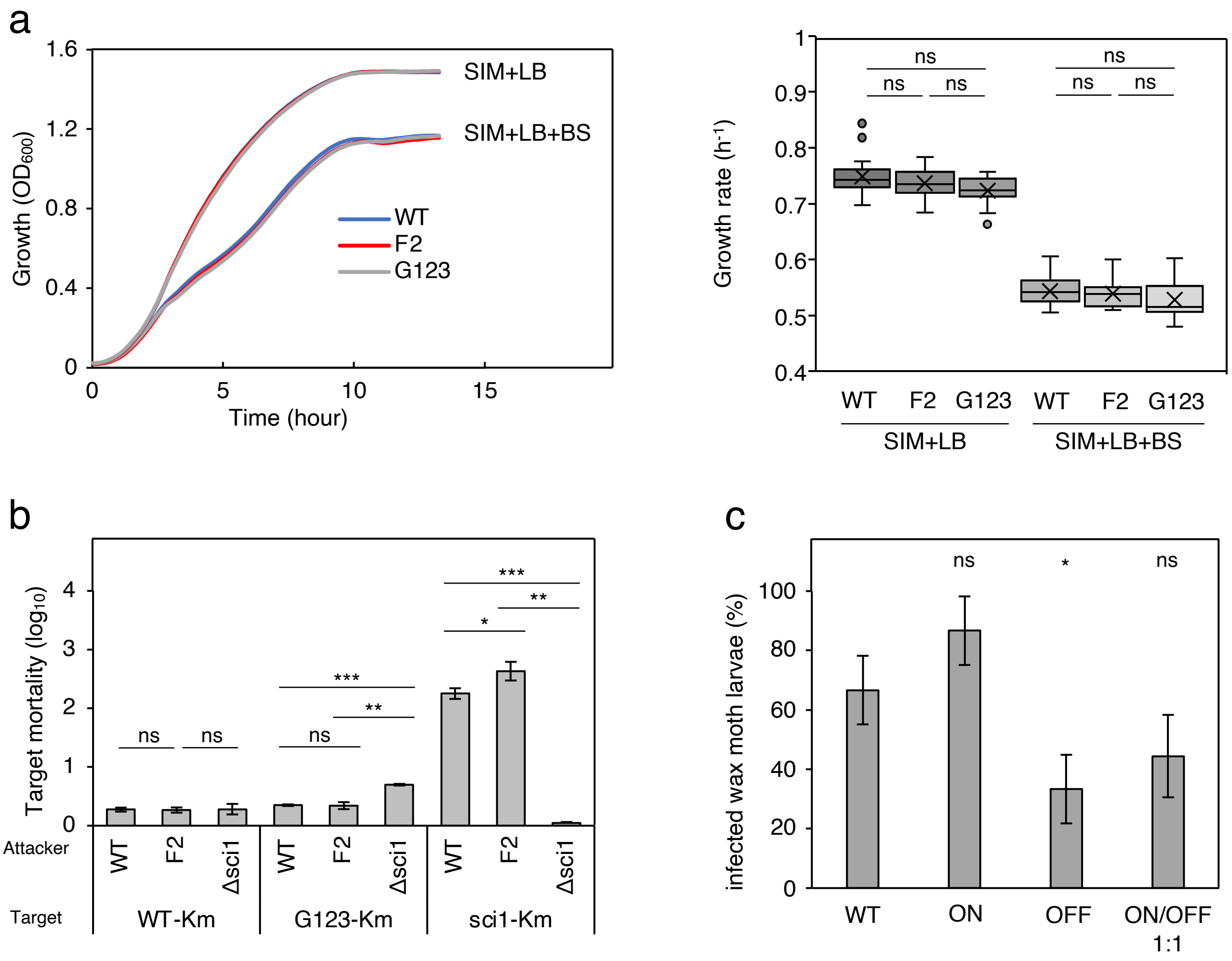

S8 Figure | Potential roles of T6SS heterogeneity. (a) Growth curve (left) and growth rate (right) of EAEC WT, F2 and G123 derivatives in SIM or SIM supplemented with bile salts (SIM+BS). The Box plot representation of the growth rates (calculated using growthcurver package in R studio [70]). The statistical analyses (Box plot representation with median values (horizontal bars), mean (crosses), 25th and 75th percentiles (lower and upper boundaries) ± SD (error bars) from 3 independent replicates) is shown on right. Statistical significance between the different strains (Student *t*-test; ns, non-significant) is indicated. **(b)** Competition assay between EAEC WT, F2 or Δ*sci1* attacker cells and EAEC WT, G123 or Δ*sci1* recipient cells carrying a kanamycin resistance cassette. Mortality was measured by the SGK method. The data represent means (vertical bars) ± SD (error bars) from 9 replicates (3 technical replicates from independent biological triplicates). Statistical significance between conditions (Student *t*-test; ns, non-significant; *, $p < 0.1$; **, $p < 0.01$; ***, $p < 0.001$) is indicated. **(c)** Infective success of EAEC WT, ON homogeneous F2 strain, OFF G123 mutant strain or a 1:1 mix of F2 and G123 in the *Galleria mellonella* host. Infective success is determined as a recovery of detectable CFU on agar plates. The data represent means (vertical bars) ± SD (error bars) from 3 independent replicates, each with 15 larvae). Statistical significance between strains (one-tailed Wilcoxon *t*-test; ns, non-significant; *, $p < 0.1$) is indicated. The data underlying this Figure can be found in S1 Data.
