# Supplementary material for: Phenotypic heterogeneity optimizes trade-offs during adaptive deployment of the type VI secretion system
Source: PLoS Biol. 2026 Jun 4;24(6):e3003838. doi: 10.1371/journal.pbio.3003838 (PMC13262931; doi:10.1371/journal.pbio.3003838)
Supplement: S9 Fig — (PDF) [file pbio.3003838.s012.pdf]

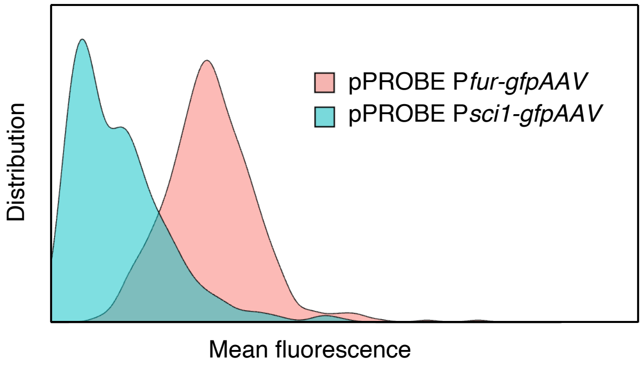

S9 Figure | P*sci1* and P*fur* activity in EAEC. Distribution profile of P*fur* (pink, homogeneous) or P*sci1* (cyan, heterogeneous) activity in EAEC WT cells carrying the pPROBE-P*fur*-*gfpAAV* or pPROBE-P*sci1*-*gfpAAV* vector, grown in SIM. The data underlying this Figure can be found in S1 Data.
