# Supplementary material for: Phenotypic heterogeneity optimizes trade-offs during adaptive deployment of the type VI secretion system
Source: PLoS Biol. 2026 Jun 4;24(6):e3003838. doi: 10.1371/journal.pbio.3003838 (PMC13262931; doi:10.1371/journal.pbio.3003838)
Supplement: Attachment — Submitted filename: 1-Taillefer-Heterogeneity-ItemizedResponsesReviewers.pdf [file pbio.3003838.s014.pdf]

Itemized responses to reviewer's comments.

Phenotypic heterogeneity optimizes trade-offs during adaptive deployment of the type VI secretion system

B. Taillefer, F. Schattenberg, T. Doan, S. Müller, E. Cascales

PBIOLOGY-D-25-03182

First, we sincerely thank the editor for handling our manuscript and the three reviewers for their positive appraisal, constructive comments and valuable suggestions, which have helped us to improve the work and the manuscript.

Reviewer #1

The type VI secretion system (T6SS) is a nanoweapon commonly used in bacterial competition. Its regulation differs between bacteria, ranging from stochastic attacks to specific activation, such as the tit-for-tat mechanism where bacteria react to attacks from neighboring bacteria with retaliatory T6SS attack. Often, T6SS are only expressed and/or activated under specific conditions. T6SS activation is energetically costly and can provoke counterattacks. In line with this, T6SS were frequently lost during evolution. Considering these points, phenotypic heterogeneity within a population could be beneficial. This manuscript studies this question. It builds on an earlier publication by the Cascales group, in which they found a DNA methylation-based, iron-dependent epigenetic switch for the enteroaggregative Escherichia coli (EAEC) sci1 T6SS gene cluster (Brunet et al., PLOS Genetics (2011), doi 10.1371/journal.pgen.1002205). The current manuscript mainly uses microscopy to determine expression and assembly of the EAEC sci1 T6SS. For both aspects, the authors observe heterogeneity within populations, with T6SS states being stable for a few generations. The study is simple, straightforward, and nicely presented. However, I have concerns about some aspects of the claims and interpretation.

Response : We thank the reviewer for the careful reading of our manuscript, the positive appraisal and for the constructive comments below that we have addressed to our best.

Major points:

1. As described above, heterogeneity of T6SS expression within clonal populations is conceptually not surprising. Heterogeneity for the assembly of the T6SS (using TssB fluorescent fusions) has been observed in many cases for different organisms. While most of these studies did not specifically investigate the heterogeneity, there are some which did. A study very directly addressing this point is Kerwien et al, PLOS Pathogens (2025), doi 10.1371/journal.ppat.1013356 (for Yersinia pseudotuberculosis), specific aspects are also shown in Lin et al, PNAS Nexus (2023), doi 10.1093/pnasnexus/pgad195 (for Vibrio fischeri, with a modeling focus) and Chen et al, Communication Biology (2025), doi 10.1038/s42003-025-08693-6 (for Yersinia enterocolitica, focusing on coregulation with other factors). The authors themselves mention several additional studies in the discussion (refs. 50-54). The manuscript under review is a valuable addition, as it additionally looks at expression specifically, the stability of heterogeneity across populations on the basis of the previously found epigenetic switch, and tries to show the role of heterogeneity in competition in a quantitative way. Nevertheless, the novelty claims in the abstract and several points in the manuscript do not appear justified in their current form.

Response : We thank the reviewer for this important comment. We fully agree that heterogeneous T6SS expression and activity have been observed in several bacterial species, including recent studies in *Yersinia* and *Vibrio*. We have therefore revised the manuscript to moderate our claims of novelty and to better position our work within the existing literature.

In the Abstract :

original version : ~~While its regulation is typically considered homogeneous across clonal populations,~~ here we report that enteroaggregative *Escherichia coli* (EAEC) exhibits stable phenotypic heterogeneity in T6SS expression.

Revised version: Fluorescent microscopy recordings have shown that T6SS expression and/or activation is heterogeneous in clonal populations of many bacterial species. However, whether T6SS heterogeneity is genetically controlled or stochastic and what is the physiological relevance of T6SS heterogeneity are yet unknown. Here we report that enteroaggregative *Escherichia coli* (EAEC) exhibits stable phenotypic heterogeneity in T6SS expression.

In the Introduction:

- Beginning of paragraph 3

Original version: Promoter architectures that couple transcription factor binding with DNA methylation can generate bistable gene expression states, producing epigenetic regulation and phenotypic heterogeneity [8]. ~~Although such regulation has not previously been described for the T6SS~~, fluorescence microscopy using translational fusions to T6SS core components has suggested heterogeneous expression in EAEC [9-15]. Phenotypic heterogeneity refers to the emergence of distinct phenotypic states within a ~~genetically identical~~ population, driven by cell-to-cell variation in gene expression [8,16-18]. Such heterogeneity can arise from genetic (e.g.,

DNA rearrangements) or non-genetic (e.g., DNA methylation, feedback loops, stochastic fluctuations) mechanisms.

Revised version: Promoter architectures that couple transcription factor binding with DNA methylation can generate bistable gene expression states, producing epigenetic regulation and phenotypic heterogeneity [8]. Indeed, fluorescence microscopy using translational fusions to T6SS core components has suggested heterogeneous expression in several species, including EAEC [9-15]. T6SS heterogeneous expression or activation has also been observed in several other species [16-22]. Phenotypic heterogeneity refers to the emergence of distinct phenotypic states within a clonal population, driven by cell-to-cell variation in gene expression [8,23-25]. Such heterogeneity can arise from genetic (e.g., DNA rearrangements) or non-genetic (e.g., DNA methylation, feedback loops, stochastic fluctuations) mechanisms. In *Yersinia pseudotuberculosis*, T6SS4 heterogeneous expression has been shown to be controlled by the heterogeneous expression of the gene encoding the RovC transcriptional regulator [21], whereas the *Pseudomonas aeruginosa* T6SS-H1 bistable expression is controlled by c-di-GMP levels via a yet unknown mechanism [22]. These observations suggest that phenotypic heterogeneity might be a recurring feature of T6SS regulation, although the underlying mechanisms can differ markedly between bacterial species.

- 2/3 of paragraph 3:

Original version: In division of labor, phenotypically distinct subpopulations accomplish specialized tasks independently that enhance group fitness [21]. Such a strategy is observed in *Myxococcus xanthus* fruiting body formation [22] and in heterogeneous expression of the *Salmonella enterica* type III secretion system (T3SS), where ON cells enable host invasion while OFF cells divide more rapidly to sustain infection [23].

Revised version: In division of labor, phenotypically distinct subpopulations accomplish specialized tasks independently that enhance group fitness [28]. The *P. aeruginosa* T6SS-H1 activity is inversely associated with expression of other virulence factors such as the T3SS, suggesting that T6SS-H1 heterogeneity can be embedded in broader physiological specialization programs [22]. Such a strategy is also observed in *Myxococcus xanthus* fruiting body formation [29] and in heterogeneous expression of the *Salmonella enterica* type III secretion system (T3SS), where ON cells enable host invasion while OFF cells divide more rapidly to sustain infection [23].

In the Discussion:

- Center of paragraph 3

Original version: Populations with a mixed ON/OFF composition thus maximize competitive advantage while avoiding excessive reciprocal killing. In polymicrobial communities such as the gut where hundreds of species cohabit, bacteria have developed antibacterial weapons but also defence mechanisms against these weapons.

Revised version: Populations with a mixed ON/OFF composition thus maximize competitive advantage while avoiding excessive reciprocal killing. Indeed, recent work has shown that that

the competitive success of T6SS-armed bacteria depends not only on killing capacity *per se*, but also on activation dynamics, weapon deployment and associated costs. In *Vibrio fisheri*, faster activation of the T6SS can provide a competitive advantage whereas excessive T6SS production can become counterproductive once its cost overweighs its benefits [52]. Our results further suggest that competition success may also depend on T6SS phenotypic heterogeneity operating as a frequency-dependent strategy that optimizes population fitness. This trade-off is particularly evident in multispecies contexts, where increasing the proportion of T6SS-active cells enhances killing but incurs a non-linear survival cost. Such dynamics may represent an evolutionarily stable strategy in polymicrobial communities. Indeed, in communities such as the gut where hundreds of species cohabit, bacteria have developed antibacterial weapons but also defence mechanisms against these weapons.

- Center of paragraph 7

Original version: T6SS heterogeneity may extend beyond EAEC. Fluorescence microscopy recordings have revealed heterogeneous T6SS expression in many species, including *Burkholderia thailandensis*, *Pseudomonas putida*, *P. fluorescens*, *Serratia marcescens*, *Salmonella enterica* Typhimurium, *Acinetobacter baylii* or *Vibrio fischeri* [50-54], despite their distinct regulatory circuits [5,6,55]. Determining the molecular basis and functional significance of heterogeneity in these systems will clarify whether it represents a conserved trade-off strategy, a division-of-labour mechanism, or a bet-hedging strategy.

Revised version: T6SS heterogeneity may extend beyond EAEC. Fluorescence microscopy recordings have revealed heterogeneous T6SS expression in many species, including *Burkholderia thailandensis*, *Pseudomonas putida*, *P. fluorescens*, *P. aeruginosa*, *Serratia marcescens*, *Salmonella enterica* Typhimurium, *Acinetobacter baylii*, *Y. pseudotuberculosis* or *Vibrio fischeri* [16-22], despite their distinct regulatory circuits [5,6,21,22,58]. Indeed, in *Y. pseudotuberculosis*, *P. aeruginosa* and EAEC, distinct regulatory architectures converge on similar heterogeneous outputs. Whereas T6SS heterogeneity in *Y. pseudotuberculosis* and *P. aeruginosa* depends on the RovC transcriptional regulator and c-di-GMP levels, respectively, our work demonstrates that EAEC T6SS heterogeneity arises from a complex interplay between Fur and DNA methylation regulations. Determining the molecular basis and functional significance of T6SS heterogeneity in other bacterial species will likely reveal the variety of regulatory mechanisms underlying heterogeneity and will clarify whether it represents a conserved trade-off strategy, a division-of-labour mechanism, or a bet-hedging strategy.

---

2. Several aspects of Figure 4, the experiment aiming to show benefits of the heterogenous expression in specific conditions, are unclear. How do the authors get and analyze the 0-100% "ON" cell fractions? Why is even the 100% "ON" fraction killed less by P. aeruginosa than the WT, whose advantage should be precisely that it is killed less due to its heterogenous T6SS expression?

Response : We used synthetic mixtures by mixing constitutive OFF state cells (G123 mutant) and constitutive ON state cells (F2 mutant) in the desired ration to have 0-100% ON cell fractions. These fractions were analyzed by cell sorting to check that the appropriate ratio has been obtained. If this trick is useful to mimic different ratios, F2 cells present a higher T6SS activity than WT cell (as shown with a better killing activity against *E. coli* W3110 - Fig. 4a). As a consequence, we speculate that the 100% ON cell population is more aggressive than the WT and may kill *P. aeruginosa* faster or more efficiently, with less retaliatory attacks, leading to a decreased mortality compared to the WT. However, we still see the importance of ON/OFF equilibriums using synthetic mixtures.

To support the claim that the heterogenous expression enhances competitiveness in near-native conditions would require stronger results, including multi-species competition assay, as depicted in Fig. 5.

Response : We thank the reviewer for this insightful suggestion. To address this point, we performed additional experiments that directly test the ecological relevance of T6SS heterogeneity in a multispecies context. The revised manuscript now introduce a three-species competition assays (new Figure 5), combining EAEC, a T6SS-negative and susceptible prey (*E. coli* W3110), and a retaliatory T6SS$^+$ competitor (*P. aeruginosa* PAO1Δ*retS*) in a 1:1:1 ratio. Figure 5a shows that with these three strains, EAEC and P. aeruginosa engage in reciprocal T6SS-mediated killing, leading to strong mortality in both populations, the decrease of EAEC hence favorising the survival of W3110. When the *P. aeruginosa* T6SS-H1 mutant is used in the 3-species competition assay, there is no retaliatory attacks, and EAEC survive better, leading to decrease in W3110 survival. When the EAEC T6SS sci1 mutant is used in the 3-species competition assay, we observed less mortality for both W3110 and *P. aeruginosa*. Finally, when the *P. aeruginosa* T6SS-H1 and EAEC T6SS sci1 mutants are used, the equilibrium between the three species is conserved. In Figure 5b, we conducted the 3-species competition assay with various ON/OFF ratios. Increasing the ratio of EAEC ON cells increases P. aeruginosa mortality but also the rate of counterattacks, leading to an increase of EAEC mortality (and as a consequence less attacks on W3110 and an increase of W3110 survival). These experiments demonstrate that T6SS heterogeneity determines population outcomes in complex communities by balancing killing efficiency and survival under retaliatory pressure.

We believe that these new data substantially strengthen our conclusions and we wish to thank again the reviewer for proposing it.

We have thus added a paragraph describing these results at the end of the Results section:

To further evaluate the ecological relevance of EAEC T6SS Sci1 heterogeneity, we performed a three-species competition assays with EAEC, the T6SS-negative and susceptible *E. coli* W3110 strain, and *P. aeruginosa* PAO1Δ*retS*, mixed in a 1:1:1 ratio. Fig. 5a shows that in this three-species competition assay, EAEC and *P. aeruginosa* engaged in reciprocal T6SS-mediated killing, leading to high mortality in both populations. Under these conditions, the strong reduction in EAEC abundance limited its ability to eliminate the susceptible W3110 strain. In contrast, when the H1-T6SS of *P. aeruginosa* was inactivated, EAEC efficiently eliminated both competitors while experiencing reduced mortality. Inactivation of both the

EAEC Sci1 T6SS and *P. aeruginosa* H1-T6SS led to a stable equilibrium of the three strains during co-incubation.

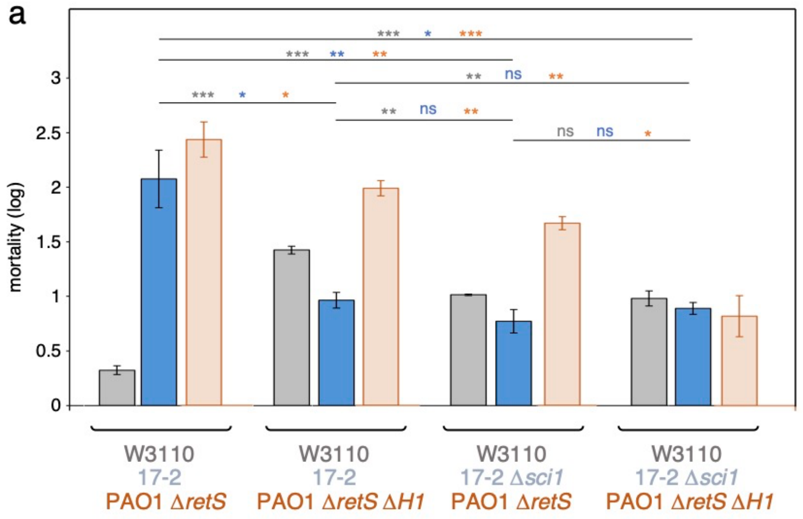

Figure 5 (a) Three-species competition assays between *E. coli* K-12 W3110 (grey), EAEC strain 17-2 (WT or Δ*sci1*, blue), and *P. aeruginosa* PAO1Δ*retS* or PAO1Δ*retS*ΔH1-T6SS (orange) at a 1:1:1 ratio. Mortality of each bacterial species was quantified by the SGK method [40].

To quantitatively assess the impact of phenotypic heterogeneity, we varied the proportion of T6SS-active (ON) cells within the EAEC population. Increasing the fraction of ON cells enhanced killing of *P. aeruginosa*, but also resulted in an increase in EAEC mortality due to retaliatory attacks (Fig. 5b). As a consequence, W3110 survival increased as EAEC populations became depleted through reciprocal killing.

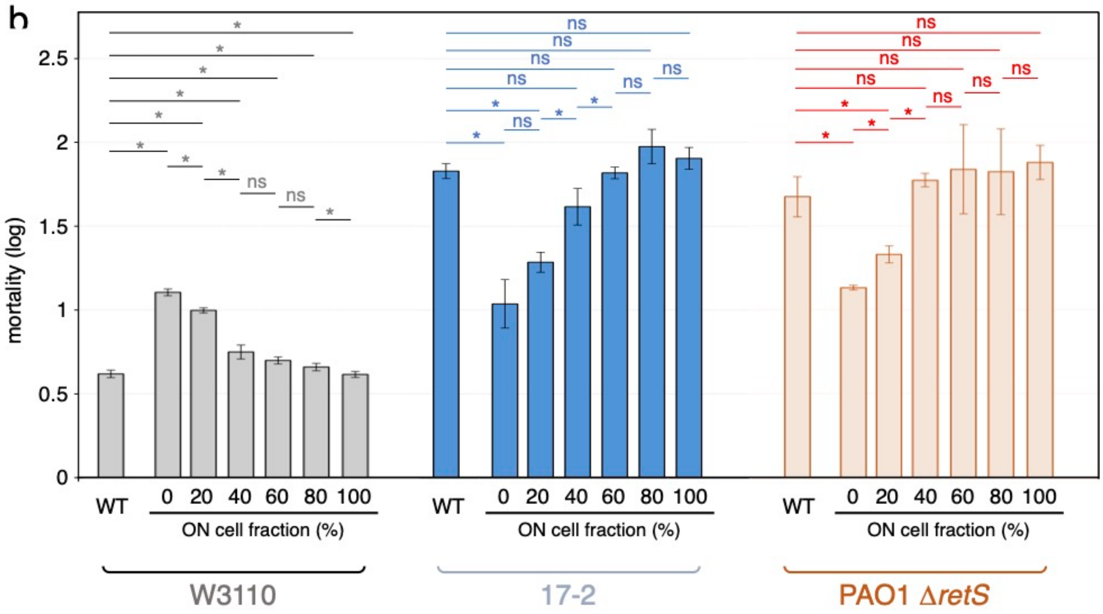

Figure 5 (b). Three-species competition assays between *E. coli* K-12 W3110 (grey), EAEC strain 17-2 (WT or increasing ON/OFF ratios (0-100%), blue), and *P. aeruginosa* PAO1Δ*retS* or PAO1Δ*retS*ΔH1-T6SS (orange) at a 1:1:1 ratio. Mortality of each bacterial species was quantified by the SGK method [40]. The data represent means (vertical bars) ± SD (error bars) from nine replicates (technical triplicates of three biological replicates). Statistical significance compared to the WT strain (one-tailed Wilcoxon's *t*-test; ns, non-significant; *, $p < 0.1$; **, $p<0.01$; ***, $p < 0.001$) is indicated with the strain color code.

Minor points:

1. Line 82. Check sentence (expression is debated?).

Response : We thank the reviewer for noticing this. The sentence has been corrected for clarity.

Original sentence:

T6SS expression, which was initially speculated to be energetically expensive for the cell, is now debated.

Revised sentence:

Although T6SS expression was initially speculated to be energetically expensive for the cell, this assumption is now debated.

---

2. Line 164. What is meant by "homogeneous ON state" in Fur box mutants? Fig. 3B shows T6SS+ rates of 45% (lower than WT) to 65% (higher, but far from 100%).

Response : “Homogeneous ON state” refers to the T6SS transcriptional activity (Figure S6a). Since the T6SS is dynamic (assembly, contraction, recycling) and it is not synchronized among all cells in the population, we never observe 100% T6SS+ cells in our fluorescence microscopy experiments. In addition, as mentioned in the first paragraph of the Results section, the use of the TssB-sfGFP translational fusion showed that there is three populations (no expression, expression but no assembly, and expression and assembly)(Figure 1a), suggesting that there is also another level of heterogeneity at the assembly stage, that we decide to not address in this work. We therefore used throught the manuscript a transcriptional fusion in which the sfGFP gene is inserted in between two genes of the T6SS sci1 gene cluster.

---

3. Lines 182-186. Is it really clear from the data (e.g. Fig. S5) that the overexpression strain (F2) also more frequently activates the T6SS? If not, the statement should be specified.

Response : Yes, the T6SS is not only overexpressed in F2 cells, but there is an significant increase of T6SS activity (Fig. 3b – panel T6SS+ frequency). We have also added a Supplemental Figure (new Figure S5) that presents a statistical analyses of the number of sheath per cell, showing that F2 cells have a significant higher number of assembled T6SS per cell compared to WT cells.

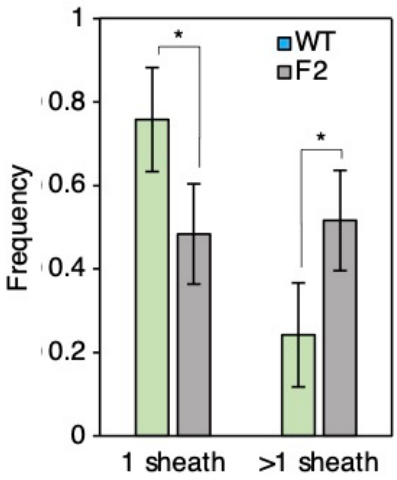

Figure S5. T6SS sheath frequency in WT and F2 TssB-GFP cells. Statistical analyses reporting the percentage of WT (blue bars) and F2 (grey bars) cells assembling one or more sheaths per cell. Only cells with dynamic sheaths were analyzed. The data represent means (vertical bars) ± SD (error bars) from 168 (WT) and 185 (F2) cells, from 8 fields from 4 biological replicates (2 fields per replicate). Statistical significance between the WT and F2 strains (one-tailed Wilcoxon's *t*-test; *, $p < 0.1$) is indicated

---

4. The "OFF" population in Fig. 2a, right panel, also seems to express the T6SS heterogeneously (albeit in a lower percentage of cells).

Response : We agree that the terms "homogeneous ON" and "homogeneous OFF" were potentially misleading and could suggest strictly uniform populations, which is not the case. In Fig. 2 we observe microcolonies formed from a single ON or single OFF cell, and we observed colonies mostly in the 'ON' of 'OFF' or 'heterogeneous' state. In Fig. S3, we track the progeny of a single ON (panel a) or OFF (panel b) cell over 7 generations in a microfluidic device. These experiments show that microcolonies derived from a single ON or OFF founder cell are predominantly composed of ON or OFF cells, respectively. In this context, the term "homogeneous" was intended to describe the dominant state of the microcolony, rather than implying that all cells are strictly identical.

To clarify this point, we now refer in the manuscript to 'predominantly OFF or ON populations' or to 'OFF-enriched' or 'ON-enriched' populations.

Original sentences : Individual founder cells gave rise to ~~homogeneous~~ OFF, ~~homogeneous~~ ON, or heterogeneous microcolonies (Fig. 2a, S3 Fig.). ~~Homogeneous~~ ON and heterogeneous colonies were more frequent than ~~homogeneous~~ OFF colonies, resulting in a population distribution similar to that observed in liquid culture (CV > 0.20; Fig. 2b).

Revised sentences : Individual founder cells gave rise to predominantly OFF, ON, or heterogeneous microcolonies after 7-10 generations (Fig. 2a, S3 Fig.). ON-enriched and heterogeneous colonies were more frequent than OFF-enriched colonies, resulting in a population distribution similar to that observed in liquid culture (CV > 0.20; Fig. 2b).

Reviewer #2

In this manuscript the authors observe significant heterogeneity in T6SS expression in enteroaggregative E.coli (EAEC). The expression of this T6SS locus has previously been shown to be under the regulatory control of the transcription factor Fur and the Dam methylase. Using nifty and innovative approaches, the authors investigate possible evolutionary reasons for heterogeneous expression. They find that heterogeneity arises from methylation dependent epigenetic switches and that although expression of T6SS does not come at a fitness cost, provoking tit-for-tat retaliation from bystander bacteria does. Cells not expressing the complete T6SS system are still protected by toxin delivery from kin-bacteria, suggesting that heterogeneous expression of the completeT6SS could be a bet-hedging strategy where a subpopulation of ON cells will protect the colony from attackers, while OFF cells are protected from tit-for-tat mediated attacks.

This is a very cool finding and interesting to the field. The manuscript is well written and adds our understanding on bacterial warfare and the burdens of toxin delivery on bacteria. However, there are a few concerns that would require attention before the manuscript could be acceptable for publication. Mainly, some controls and statistical analyses are lacking, and some textual /illustrational modification would also benefit the manuscript. See the main and minor comments below for more detail.

Response : We thank the reviewer for the positive assessment of our work and for the constructive and detailed comments. We agree that several aspects of the manuscript required clarification and strengthening, in particular regarding controls, statistical analyses, and data presentation. We have revised the manuscript accordingly and performed additional analyses where necessary. Below, we address each point in detail.

Main comments:

1. One of the main arguments of the above theory is that the OFF population is protected from kin-delivery through expression of the TliI antitoxin from a different promoter inside the T6SS locus. The authors test this by competing WT, ON (F2) and deltasci1 inhibitors against WT and OFF (G123) targets. However, in Fig S2 only WT targets are shown. The data with the OFF targets mentioned in the text should be included in the actual figure.

Response : We thank the reviewer for noticing this. We have performed the competition assay using Wild-type, G123 (OFF) and Δ*sci1* (lacking the whole T6SS gene cluster) recipients against WT, F2 (ON) and Δ*sci1* attacker cells. The results, now included in the revised version of the manuscript (Figure S8B), demonstrate that the Δ*sci1* recipients are susceptible to WT and F2 attacks, whereas the G123 cells are not. This result supports the hypothesis that there is

a basal level of immunity production when the main promoter is mutated, which is in agreement with the existence of an internal promoter genes upstream the gene encoding the immunity (Brunet et al., 2021) and that this basal immunity production is sufficient to confer protection The description of the results in the Results section has been clarified.

Original sentence: ~~However,~~ we did not detect~~ed~~ kin killing in EAEC: competition assays showed that the presence of the ON-homogeneous F2 strain did not increase mortality of WT or of G1G2G3 OFF cells (S7b Fig.), ~~likely owing to~~ basal expression of the immunity gene *tli1* from the internal promoter P_{4532} [34].

Revised sentence: We did not detect kin killing in EAEC: contrarily to Δ*sci1* mutant cells (i.e., deleted of the whole T6SS gene cluster) that are susceptible to WT attacks, G1G2G3 cells were not (S8b Fig.). In addition, competition with the ON-homogeneous F2 strain did not increase mortality of WT or of G123 OFF cells (S8b Fig.). This result suggests that basal expression of the immunity gene *tli1* from the internal promoter P_{4532} [34] is sufficient to confer protection against kin ON cells.

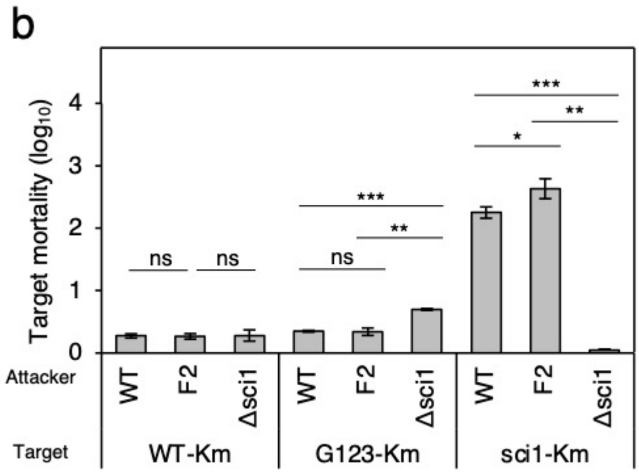

S8 (b) Figure. Competition assay between EAEC WT, F2 or Δ*sci1* attacker cells and EAEC WT, G123 or Δ*sci1* recipient cells carrying a kanamycin resistance cassette. Mortality was measured by the SGK method. The data represent means (vertical bars) ± SD (error bars) from 9 replicates (3 technical replicates from independent biological triplicates). Statistical significance between conditions (Student *t*-test; ns, non-significant; *, $p < 0.1$; **, $p < 0.01$; ***, $p < 0.001$) is indicated

---

It is also not clear why these experiments where performed at a 1:4 ratio, rather than the 4:1 ration used in Fig. 3C. Using a lower ratio could falsely indicate that there is protection against kin-bacteria, in particular as there is no control included where inhibition actually occurs. This data is vital for the model to work. Therefore, the experiment should be repeated at a 4:1 ratio, including a control showing that indeed T6SS mediated killing does occur in this context. For proper statistical analysis and to align better with the rest of the experimnets, more than 2 bioreps should be used.

Response : Throughout the manuscript, all quantitative SGK assays have been performed with the 4:1 attacker:target ratio whereas all qualitative CPRG (LAGA) assays have been performed with the 1:4 attacker:ratio because the signal of β-galactosidase release is not sufficient for proper staining of the colony with a 4:1 ratio. However, using these optimized methods, we always obsevred a strict correlation between the qualitative (CPRG) and quantitative (SGK)

assays. Regarding the bioreps, all assays have been done, at least, with three biological replicates.

---

2. The second important point that the authors make is that the ON/OFF state is determined by a Dam-methylation dependent epigenetic switch. I agree that this is likely, but the data in figure S4C lacks statistical analysis, making it difficult to assess whether the sorted ON colonies indeed stay ON etc. Anova with posthoc test would tell if this is indeed the case. Also, discussion about the switch-rates for ON/OFF would be interesting and could be calculated on the same data.

Response : In Figure S4, the orange circles represent T6SS+ cell frequency in a microcolony grown from an OFF cell whereas grey circles represent T6SS+ cell frequency in a microcolony grown from a ON cell. The OFF or ON dashed lines represent the average T6SS+ cell frequency in micro-colonies grown from OFF or ON cells, respectively. For clarity, and as suggested by the reviewer, we have now performed appropriate statistical analyses on these datasets, which are presented in Figure S4d. Specifically, we used statistical analyses to assess differences between ON- and OFF-derived populations over time. These analyses confirm that sorted ON and OFF populations maintain their respective states over short timescales and converge toward the equilibrium distribution over longer timescales.

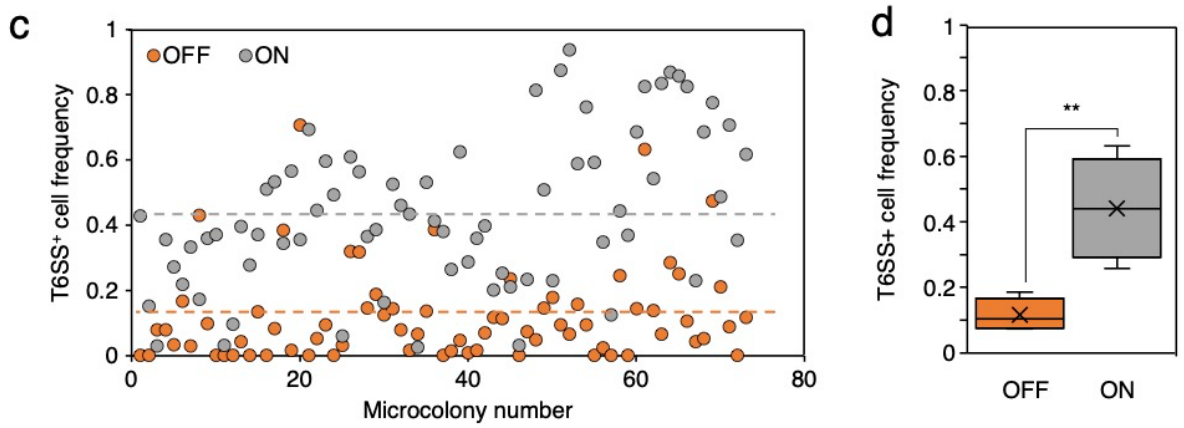

S4 Figure (c) T6SS$^+$ cell frequency in microcolonies from single-cell experiments of sorted ON and OFF subpopulations. Each dot is a microcolony. The overall average frequency is shown as a dotted line with the corresponding colour. More than 3,400 cells were analysed from 73 microcolonies from 6 independent replicates. The statistical analyses (Box plot representation with median values (horizontal bars), mean (crosses), 25th and 75th percentiles (lower and upper boundaries) ± SD (error bars) from n=6 cells) is shown in panel **(d)**. Statistical significance compared between the two populations (one-tailed Wilcoxon's t-test; **, p<0.01) is indicated.

Regarding the suggestion to estimate switching rates, we agree that this is an interesting aspect. However, precise estimation of switching rates would require dedicated time-resolved lineage

tracking or modeling approaches that we were not able to perform in the frame of this project. We have therefore included a sentence in the Discussion acknowledging this point as an important direction for future work.

---

3. In general, statistics seem to be missing in many figures: Fig 3b, Fig 4abc, Fig S7d. Please add the appropriate statistical analyses to the figures. In addition, please provide the raw-data for the experiments as a supplementary excel file.

Response : We thank the reviewer for highlighting this issue. We agree that the initial version of the manuscript lacked sufficient statistical support in several figures.We now provide statistical analyses for all figures. The statistical tests used (Student's t-test, Wilcoxon test for non-parametric data, and ANOVA where appropriate) are now indicated in the figure legends and a dedicated "Statistical analysis" section has been added to the Materials and Methods. Finally, Raw numerical data used to generate all figures are now be provided as supplementary datasets.

---

4. The assay used for enumerating target survival in figs 4ab and S7b is somewhat untraditional. It is unclear how the authors quantify survival from the hours it takes for the culture to grow to a certain OD600, when no standard curve is shown. Please include a standard curve and maybe an overview figure for this method to make it more clear.

Response : We agree that the SGK method may not be immediately intuitive. We have revised the Results and Methods sections to more clearly explain the principle of the SGK assay. In particular, we now explicitly state that the time required for the recipient population to resume exponential growth is inversely correlated with the initial number of surviving cells, and the values reported are used as a proxy for survival. As the technical paper reporting this method, which has an overview figure of the SGK assay, is cited in our manuscript, we hope that the referee will understand that we decided not to add an overview figure here.

In the SGK experiments reported in our manuscript, we did not use a standard curve to calculate the initial number of bacteria surviving after the competition assay, but only report the mortality in log-difference scale. Therefore our data do not report "how much cells are killed" but rather "what is the difference of mortality between conditions (i.e., different attackers)".

Results section

Originial text : We next considered a suicide model in which OFF cells act as a nutritional reservoir for ON cells. In this case, OFF cells will not produce the Tli1 immunity protein and therefore can be killed by ON cells to support their growth. However, we did not detect kin killing in EAEC.

Revised text: We next considered a suicide model in which OFF cells act as a nutritional reservoir for ON cells. In this case, OFF cells will not produce the Tli1 immunity protein and therefore can be killed by ON cells to support their growth. To test this hypothesis, we conducted competition experiments using the SGK assay [40]. The SGK assay is a growth recovery method that measures the time necessary for surviving bacteria to reach mid-exponential phase, which is inversely proportional to the initial number of surviving cells. We did not detect kin killing in EAEC:…

Material and methods section

Original text: For quantitative SGK assays, […]. Recipient survivor growth was monitored using a TECAN microplate reader. Growth parameters were extracted with the R package growthcurver [65]. Because the time to resume growth depends on the initial cell concentration, the mid exponential time point (Tmid) was used as a proxy for recipient mortality.

Revised text: For quantitative SGK assays, […]. Recipient survivor growth was monitored using a TECAN microplate reader. Growth parameters were extracted with the R package growthcurver [68]. Because the time to resume growth depends on the initial cell concentration (i.e., the time required for the recipient population to resume exponential growth is inversely correlated with the initial number of surviving cells), the mid exponential time point (Tmid) was used as a proxy for recipient mortality.

---

4. There seems to be a loss in virulence/colonization ability for the OFF mutants in the Galleria mellonella assay. This should at least be mentioned in the results/discussion as this is rather cool.

Response : We thank the reviewer for pointing out this observation. While we did not want to emphasize too much this result to be more focused on the heterogeneity in the context of polymicrobial communities, we agree that the reduced infective success of the OFF mutant is noteworthy and that it deserves to be discussed. We have therefore added a short discussion of this Discussion section:

The heterogeneity strategy described may enhance colonization success by combining offensive capacity with reduced susceptibility to retaliation. By modulating the fraction of ON cells, EAEC limits the activation of costly defensive strategies, representing an indirect form of adaptive defence. This ON/OFF balance may, however, come at a cost in other biological contexts. In particular, while OFF cells may be advantageous in interbacterial competition by limiting exposure to retaliatory attacks, they may be less effective in host-associated environments where T6SS activity contributes to virulence or niche establishment. Indeed, our results showed that ON cells better colonize the wax moth model whereas OFF cells are less

efficient. This context-dependent trade-off suggests that phenotypic heterogeneity may allow bacterial populations to balance competing selective pressures depending on the ecological conditions.

Minor comments:

1. Skip the B-GFP, C-GFP-K abbreviations throughout the manuscript. They are mainly used in the figures and just confuse the reader. There is plenty of space to write out the full name of the fusion in the figures, which would make the manuscript much easier to read.

Response : We have modified the terminology in the text and in the figures as suggested, by using descriptive names : TssB-GFP instead of B-GFP, and TssC-GFP-TssK instead of C-GFP-K.

---

2. Figure 2 legend: "Short, medium and long: frequencies after ~5, ~15 and ~20 generations, 622 respectively." Write the number of generations in the graph instead of long, medium, short. The terms long, medium, short are unclear and could be reffering to e.g. cell length instead.

Response : As recommended by the reviewer, we have changed the terms “short”, “medium”, and “long” with a regular axis with the number of generations

---

3. Line 45. "…ts.." - meaning not clear. Should this say T6SS?

Response : The typo (its) was corrected.

---

4. Line 111: "These observations suggest that heterogeneity occurs at both the transcriptional/translational level (expression/production vs no expression/production) and the post-translational level (assembly vs. no assembly)"-This statement requires some controls: i.e. a strain lacking T6SS and a strain unable to assemble T6SS to show that what you observe in the images are indeed T6SS foci and lack of the same.

Response : A control for following transcription/translation of the TssC-GFP-TssK reporter has been used at the very start of our project, notably for defining the parameters for fluorescence microscopy recordings (e.g., to discard autofluorescence using optimized acquisition time, etc). For the T6SS foci using the TssB-sfGFP fusion, we have been working daily with this reporter for 14 years, and we have the pretention to think that we know quite well how it behaves. ☺

---

5. Line 133: Individual founder cells gave rise to homogeneous OFF, homogeneous ON, or

heterogeneous microcolonies. - How were the different populations determined? What cut-offs of fluorescence was used? Maybe illustrate this in the histograms of Figure 1ab.

Response : For the transcriptional TssC-GFP-TssK fusion, the OFF/ON cut-off was defined by the level of autofluorescence of EAEC cells that do not express the GFP. For the translational TssB-GFP fusion, the OFF/ON cut-off was determined empirically, by imaging the sorted populations and determining the parameters for which >95 of the sorted ON cells presented sheaths. The cut-offs are now indicated in Figure 1ab.

---

6. Line 134: Homogeneous ON and heterogeneous colonies were more frequent than homogeneous OFF colonies, resulting in a population distribution similar to that observed in liquid culture (CV > 0.20; Fig. 2b) - Data for quantification of ON and OFF colonies is not shown. Etiher remove this statement or add the data.

Response : Figure 1c shows that the coefficient of variation (CV) of a population grown in a liquid culture is comprised between 0.2 and 0.3. By analyzing microcolonies grown from individual cells, we measured a CV of 0.24 (Figure 2b). This observation suggests that while microcolonies can form homogeneous or heterogeneous populations, the overall population (i.e., combining all microcolonies) is heterogeneous, with a CV similar to that observed with a liquid culture population.

---

7. Line 136: "The overall frequency of T6SS$^+$ cells, which was lower in microfluidics, likely due to slower growth under reduced oxygen availability compared with liquid conditions" -Odd sentence. Please rephrase.

Response : We have rephrased this sentence to improve clarity.

Original sentence : The overall frequency of T6SS$^+$ cells~~, which~~ was ~~lower~~ in microfluidics, likely ~~due to~~ slower growth ~~under reduced~~ oxygen availability compared with liquid ~~conditions~~ (Fig. 2b).

Revised sentence : The overall frequency of T6SS$^+$ cells was reduced in microfluidics, likely reflecting slower growth associated with lower oxygen availability compared with liquid cultures (Fig. 2b).

---

8. Line 206. Please state why you used a strain with a deltaretS. This might not be clear for the non-expert readers.

Response : We have added a brief phenotypic description of the PAO1 Δ*retS* strain.

Original sentence : However, when competing with ~~the defensive T6SS+ strain~~ *Pseudomonas aeruginosa* PAO1Δ*retS*, increasing the ON/OFF ratio led to higher EAEC mortality (Fig. 4b).

Revised sentence: However, when competing with the model retaliatory T6SS-positive competitor *Pseudomonas aeruginosa* PAO1Δ*retS* (i.e., a strain that constitutively expresses the H1-T6SS but only assembles it and fires upon attacks from competitors [48]), increasing the ON/OFF ratio led to higher EAEC mortality (Fig. 4b).

---

9. "Fig. 3c shows that the T6SS activity correlated with the proportion of T6SS$^+$ cells: G1 and G2 mutants exhibited reduced killing, while G3 and G123mutants were inactive under inducing conditions (Fig. 3c)." .Not shown in Fig 3c. G1 and G3 look identical. G2 looks like wt and G123 looks OFF. Please rephrase or show quantitative data if you want to claim this.

Response : We agree that the original description did not accurately reflect the data. We have revised the text to better match the observed patterns.

Original sentence: Fig. 3c shows that the T6SS activity correlated with the proportion of T6SS$^+$ cells: ~~G1 and~~ G2 mutants exhibited reduced killing, while G3 and G123 mutants were inactive under inducing conditions (Fig. 3c).

Revised sentence: Fig. 3c shows that the T6SS activity roughly correlated with the proportion of T6SS$^+$ cells: G2 mutant cells exhibited reduced killing, while G1 and G3 cells were significantly impaired in killing and G123 mutant cells were inactive under inducing conditions (SIM, Fig. 3c).

Also, please include an illustration or text in the figure (not only in the legend) explaining how the color change illustrates viability of targets.

Response : We have clarified the colorimetric readout in the figure by adding a heatmap of the killing efficiency on right of Figure 3c to help the reader.

---

10. There is something odd with the peaks in figure S6ab. The peaks are either cut off at random values or there were very few data points recorded for e.g. G123 and G3 in a and G1, G2, G3 and G123 in b. Please fix this.

Response : The apparent truncation of the peaks does not reflect a lack of data points but results from a deliberate choice in data visualization. Specifically, the y-axis scale was adjusted to avoid compressing distributions with lower peak values, thereby allowing a clearer comparison of the overall distribution shapes in the different conditions.To address this concern and improve transparency, we have now included the full, non-truncated distributions as inset

panels in Figure S6ab (now Figure S7ab), as well as in Figure S2. These insets confirm that the apparent truncation is purely graphical and does not affect the interpretation of the data. We have also clarified this point in the figure legend.

Reviewer #3

In this manuscript, Taillefer and colleagues focus on phenotypic heterogeneity of the T6SS. Although I would say that this phenomenon could be observed in microscopy images of existing studies already, I agree that there has been little work entirely dedicated to phenotypic heterogeneity of the T6SS. This manuscript is very timely. The authors use an array of techniques, ranging from sophisticated microscopy to cell sorting, genetics, in vivo infection experiments, and bacterial competition assays to precisely quantify the ratio of cells with and without a T6SS sheath in a population, demonstrate that ON and OFF states are reversible, decipher the mechanism that controls the ON and OFF state, and assess the role of phenotypic heterogeneity for killing others and for being attacked. These findings shed light on a topic that has received little attention so far in studies on T6SS activity and provides important data in the ongoing debate on the costs and benefits of the T6SS.

I therefore see the results of broad interest to the fields of the T6SS, phenotypic heterogeneity, and the evolution of trade-offs.

In my opinion, the experimental work is done very thoroughly, the manuscript is clearly written and the data is well presented.

Response : We thank the reviewer for the positive evaluation of our manuscript.

I have a few comments that I would please ask the authors to consider during their revision.

Major comments

1. Use of the term "homogeneous ON" and "homogeneous OFF". Could the authors at least clarify, please. As I understand, S3 Fig. shows examples of what is referred to as "homogeneous ON" and "homogeneous OFF" in l. 133. I see the strong difference between the two, I just think it is important that the ones considered as ON are actually not all ON but some do not have the sheath, isn't it? Also among the ones that are OFF, there seems to be at least one cell that is ON, or am I missing something here?

Response : We thank the reviewer for this important comment and for the careful examination of Fig. S3. We agree that the terms “homogeneous ON” and “homogeneous OFF” were potentially misleading and could suggest strictly uniform populations, which is not the case.

In Fig. S3, we track the progeny of a single ON (panel a) or OFF (panel b) cell over multiple generations in a microfluidic device. These experiments show that microcolonies derived from a single ON or OFF founder cell are predominantly composed of ON or OFF cells, respectively. In this context, the term “homogeneous” was intended to describe the dominant state of the microcolony, rather than implying that all cells are strictly identical.

Importantly, as correctly pointed out by the reviewer, not all cells classified as ON display an assembled T6SS sheath at a given time. This reflects an additional level of heterogeneity at the level of T6SS assembly, which is known to be dynamic and temporally regulated. Conversely, OFF populations may occasionally contain rare cells exhibiting T6SS activity.

To clarify this point, we now refer in the manuscript to ‘predominantly OFF or ON populations’ or to ‘OFF-enriched’ or ‘ON-enriched’ populations.

Original sentences : Individual founder cells gave rise to ~~homogeneous~~ OFF, ~~homogeneous~~ ON, or heterogeneous microcolonies (Fig. 2a, S3 Fig.). ~~Homogeneous~~ ON and heterogeneous colonies were more frequent than ~~homogeneous~~ OFF colonies, resulting in a population distribution similar to that observed in liquid culture (CV > 0.20; Fig. 2b).

Revised sentences : Individual founder cells gave rise to predominantly OFF, ON, or heterogeneous microcolonies after 7-10 generations (Fig. 2a, S3 Fig.). ON-enriched and heterogeneous colonies were more frequent than OFF-enriched colonies, resulting in a population distribution similar to that observed in liquid culture (CV > 0.20; Fig. 2b).

This makes me very generally wondering about the idea of the competition experiments, where cells are mixed at a certain ON and OFF ratio. How much do we know about the actual ratio of bacteria with and without a functional T6SS in these spots? Isn't "ON" rather a population of bacteria with a high percentage of bacteria with a T6SS formed and "OFF" rather a population of bacteria with a low percentage of bacteria with a T6SS formed? If so, are the terms 'ON' and 'OFF' and 'homogeneous' potentially slightly misleading? In the context of this comment, it might also be worth reconsidering the graphical depiction in Fig. 5.

Response : Because wild-type ON and OFF subpopulations relax back to an equilibrium distribution within approximately 15-20 generations, it is not possible to maintain defined ON/OFF ratios over the timescale of competition assays using sorted populations alone. To overcome this limitation, we used genetically stabilized strains to generate synthetic ON/OFF ratios: the F2 strain, which is locked in a constitutive ON-like state, and the G123 strain, which is locked in a constitutive OFF-like state. These strains allow us to impose controlled and stable population-level differences in T6SS activity. The effective ON/OFF composition of these

mixtures were verified prior to each assay by assessing the proportion of T6SS-expressing cells using fluorescence microscopy and flow cytometry.

---

2. I encourage the authors to expand a bit more on existing data in other species of relevance to T6SS phenotypic heterogeneity (e.g. PMID: 40991669, PMID: 40819180). I understand that the work in the manuscript is on EAEC, do the authors expect such heterogeneity in the T6SS across species? Does existing T6SS microscopy data suggest similar phenotypes in other species?

Response : We fully agree that heterogeneous T6SS expression and activity have been observed in several bacterial species, and this was already noticed in the Discussion section of the original manuscript. A recent study on the *Yersinia* T6SS provided more details on the regulatory mechanism controling T6SS heterogeneity. We have therefore revised the manuscript to moderate our claims of novelty and to better position our work within the existing literature.

In the Abstract :

original version : ~~While its regulation is typically considered homogeneous across clonal populations,~~ here we report that enteroaggregative *Escherichia coli* (EAEC) exhibits stable phenotypic heterogeneity in T6SS expression.

Revised version: Fluorescent microscopy recordings have shown that T6SS expression and/or activation is heterogeneous in clonal populations of many bacterial species. However, whether T6SS heterogeneity is genetically controlled or stochastic and what is the physiological relevance of T6SS heterogeneity are yet unknown. Here we report that enteroaggregative *Escherichia coli* (EAEC) exhibits stable phenotypic heterogeneity in T6SS expression.

In the Introduction:

- Beginning of paragraph 3

Original version: Promoter architectures that couple transcription factor binding with DNA methylation can generate bistable gene expression states, producing epigenetic regulation and phenotypic heterogeneity [8]. ~~Although such regulation has not previously been described for the T6SS~~, fluorescence microscopy using translational fusions to T6SS core components has suggested heterogeneous expression in EAEC [9-15]. Phenotypic heterogeneity refers to the emergence of distinct phenotypic states within a ~~genetically identical~~ population, driven by cell-to-cell variation in gene expression [8,16-18]. Such heterogeneity can arise from genetic (e.g., DNA rearrangements) or non-genetic (e.g., DNA methylation, feedback loops, stochastic fluctuations) mechanisms.

Revised version: Promoter architectures that couple transcription factor binding with DNA methylation can generate bistable gene expression states, producing epigenetic regulation and phenotypic heterogeneity [8]. Indeed, fluorescence microscopy using translational fusions to T6SS core components has suggested heterogeneous expression in several species, including EAEC [9-15]. T6SS heterogeneous expression or activation has also been observed in several other species [16-22]. Phenotypic heterogeneity refers to the emergence of distinct phenotypic states within a clonal population, driven by cell-to-cell variation in gene expression [8,23-25]. Such heterogeneity can arise from genetic (e.g., DNA rearrangements) or non-genetic (e.g., DNA methylation, feedback loops, stochastic fluctuations) mechanisms. In *Yersinia pseudotuberculosis*, T6SS4 heterogeneous expression has been shown to be controlled by the heterogeneous expression of the gene encoding the RovC transcriptional regulator [21], whereas the *Pseudomonas aeruginosa* T6SS-H1 bistable expression is controlled by c-di-GMP levels via a yet unknown mechanism [22]. These observations suggest that phenotypic heterogeneity might be a recurring feature of T6SS regulation, although the underlying mechanisms can differ markedly between bacterial species.

- 2/3 of paragraph 3:

Original version: In division of labor, phenotypically distinct subpopulations accomplish specialized tasks independently that enhance group fitness [21]. Such a strategy is observed in *Myxococcus xanthus* fruiting body formation [22] and in heterogeneous expression of the *Salmonella enterica* type III secretion system (T3SS), where ON cells enable host invasion while OFF cells divide more rapidly to sustain infection [23].

Revised version: In division of labor, phenotypically distinct subpopulations accomplish specialized tasks independently that enhance group fitness [28]. The *P. aeruginosa* T6SS-H1 activity is inversely associated with expression of other virulence factors such as the T3SS, suggesting that T6SS-H1 heterogeneity can be embedded in broader physiological specialization programs [22]. Such a strategy is also observed in *Myxococcus xanthus* fruiting body formation [29] and in heterogeneous expression of the *Salmonella enterica* type III secretion system (T3SS), where ON cells enable host invasion while OFF cells divide more rapidly to sustain infection [23].

In the Discussion:

- Center of paragraph 3

Original version: Populations with a mixed ON/OFF composition thus maximize competitive advantage while avoiding excessive reciprocal killing. In polymicrobial communities such as the gut where hundreds of species cohabit, bacteria have developed antibacterial weapons but also defence mechanisms against these weapons.

Revised version: Populations with a mixed ON/OFF composition thus maximize competitive advantage while avoiding excessive reciprocal killing. Indeed, recent work has shown that that the competitive success of T6SS-armed bacteria depends not only on killing capacity *per se*, but also on activation dynamics, weapon deployment and associated costs. In *Vibrio fisheri*, faster activation of the T6SS can provide a competitive advantage whereas excessive T6SS

production can become counterproductive once its cost overweighs its benefits [52]. Our results further suggest that competition success may also depend on T6SS phenotypic heterogeneity operating as a frequency-dependent strategy that optimizes population fitness. This trade-off is particularly evident in multispecies contexts, where increasing the proportion of T6SS-active cells enhances killing but incurs a non-linear survival cost. Such dynamics may represent an evolutionarily stable strategy in polymicrobial communities. Indeed, in communities such as the gut where hundreds of species cohabit, bacteria have developed antibacterial weapons but also defence mechanisms against these weapons.

- Center of paragraph 7

Original version: T6SS heterogeneity may extend beyond EAEC. Fluorescence microscopy recordings have revealed heterogeneous T6SS expression in many species, including *Burkholderia thailandensis*, *Pseudomonas putida*, *P. fluorescens*, *Serratia marcescens*, *Salmonella enterica* Typhimurium, *Acinetobacter baylii* or *Vibrio fischeri* [50-54], despite their distinct regulatory circuits [5,6,55]. Determining the molecular basis and functional significance of heterogeneity in these systems will clarify whether it represents a conserved trade-off strategy, a division-of-labour mechanism, or a bet-hedging strategy.

Revised version: T6SS heterogeneity may extend beyond EAEC. Fluorescence microscopy recordings have revealed heterogeneous T6SS expression in many species, including *Burkholderia thailandensis*, *Pseudomonas putida*, *P. fluorescens*, *P. aeruginosa*, *Serratia marcescens*, *Salmonella enterica* Typhimurium, *Acinetobacter baylii*, *Y. pseudotuberculosis* or *Vibrio fischeri* [16-22], despite their distinct regulatory circuits [5,6,21,22,58]. Indeed, in *Y. pseudotuberculosis*, *P. aeruginosa* and EAEC, distinct regulatory architectures converge on similar heterogeneous outputs. Whereas T6SS heterogeneity in *Y. pseudotuberculosis* and *P. aeruginosa* depends on the RovC transcriptional regulator and c-di-GMP levels, respectively, our work demonstrates that EAEC T6SS heterogeneity arises from a complex interplay between Fur and DNA methylation regulations. Determining the molecular basis and functional significance of T6SS heterogeneity in other bacterial species will likely reveal the variety of regulatory mechanisms underlying heterogeneity and will clarify whether it represents a conserved trade-off strategy, a division-of-labour mechanism, or a bet-hedging strategy.

Minor comments

l. 28 "deliver" should probably read "delivers"

Response : Corrected.

---

l. 45 typo "its"?

Response : Corrected.

l. 65 just a thought and up to the authors, I personally struggle a bit with „within a genetically identical population". Aren't bacterial populations (even an overnight culture of the WT strain) rather genetically diverse? I guess they can be genetically diverse without being a problem for the story, as long as the genetic differences are not relevant for the particular trait of interest, here T6SS activity.

Response : We thank the reviewer for this remark. We agree that bacterial populations may accumulate genetic variation over time. We have slightly rephrased this sentence to clarify that we refer to clonal populations rather than genetically identical populations.

Fig. 1a, I might have missed this, is a brightfield image provided?

Response : For all microscopy experiments, we collected brightfield and fluorescence images, but for simplicity, the brightfield images were not shown in the original figures. We now show in Figure 1 images with merged bright and fluorescence fields.

How else do the authors conclude on the first of the three subpopulations (cells that do not produce TssB-sfGFP). Did the authors somehow validate their method to reliably detect cells with the TssB tube?

Response : The identification of TssB-sfGFP structures as T6SS sheaths is based on previously validated criteria, including fluorescence intensity and morphology, as described in earlier studies. To validate the detection of T6SS sheath-positive cells using the TssB-sfGFP reporter, we established a calibration procedure linking flow cytometry signals to direct visualization by fluorescence microscopy. We defined fluorescence thresholds for ON ($T6SS^+$) and OFF ($T6SS^-$) populations based on the distribution of GFP intensity measured by flow cytometry. Cells from these gated populations were then sorted and systematically examined by fluorescence microscopy. This analysis confirmed that cells classified as ON by flow cytometry predominantly displayed clearly identifiable TssB-sfGFP sheath structures, whereas cells classified as OFF lacked visible sheaths.

Might be worth checking the figure legend again, I wasn't sure about the references to left and right.

Response : For the right/left, we thank the reviewer for pointing this out. This was an error in the figure legend, and the references to “left” and “right” were indeed inadvertently reversed. We have now corrected the legend accordingly.

Fig. 3c, could the authors please indicate if these are representative images of multiple replicates?

Response : We have clarified in the figure legend that the images shown are representative of at least three independent biological experiments, each with technical triplicates.

Fig. 4 and respective text (l. 200f), I have multiple comments or thoughts here:
- I would be interested in what the ticks on the x-axis refer to, I guess it is steps of 20% but wasn't sure. Could the actually numbers be added in addition to the triangle?

Response : We agree that the *x*-axis was lacking clarity. We have now indicated the ratio (% of ON cells) on both panels.

- How do the authors explain that the WT resembles a phenotype of ON in Fig. 4b? isn't this unexpected given the idea of phenotypic heterogeneity and expected 60% ON based on the data in Fig. 1? Also, how come that the WT resembles phenotypic data of different on/off ratios in Fig. 4a and 4b?

Response : We have reported here the experimental data and we do not have definitive answer to this discrepancies. However, the fraction of transcriptionally ON cells does not directly correspond to the fraction of cells with an active T6SS. Indeed, using the TssB-sfGFP reporter, we observe that only about half of the transcriptionally ON cells assemble a T6SS sheath at a given time. As a result, a population composed of 100% transcriptional ON cells likely corresponds to only ~50% of cells with an active T6SS, whereas the WT population should contain ~30% of cells with an active T6SS.

In addition, in conditions involving a retaliatory competitor (Fig. 4b), T6SS-active cells are preferentially targeted and eliminated, which further reduces their contribution over time.

- Just a side note, isn't it really interesting that there seems to be a linear relationship between the on/off ratio and killing others, and a non-linear relationship between the on/off ratio and getting killed by P. aeruginosa? In other words, there are certain ratios that could provide a disproportionally higher gain from killing others than becoming costly when mixed with an attacker that does tit-for-tat.

Response : We agree that our data suggest a difference in how killing efficiency and susceptibility to retaliation scale with the ON/OFF ratio. In particular, killing of susceptible targets appears to increase approximately proportionally with the fraction of ON cells, whereas susceptibility to retaliation by *P. aeruginosa* shows a more non-linear response. A likely

explanation is that killing of susceptible target cells depends primarily on the number of T6SS-active cells, leading to an approximately additive effect. In contrast, retaliatory interactions involve reciprocal killing between T6SS-active cells, which can amplify cell loss and result in a disproportionate cost as the fraction of ON cells increases.

We have added a sentence in the Discussion section to highlight this point.

Original sentences: Functionally, ON cells confer antibacterial activity, whereas OFF cells do not contribute to killing but instead mitigate attacks from retaliatory T6SS^{+} competitors. EAEC T6SS heterogeneity represents a trade-off that solves a conflict between two opposing pressures: the need for ON cells to eliminate susceptible recipients, and the cost of provoking counterattacks from armed competitors.

Revised sentences: Functionally, ON cells confer antibacterial activity, whereas OFF cells do not contribute to killing but instead mitigate attacks from retaliatory T6SS^{+} competitors. Interestingly, our data suggest that killing efficiency scales approximately with the fraction of T6SS-active cells, whereas susceptibility to retaliatory killing displays a more non-linear dependence, suggesting that costs can increase disproportionately at high ON fractions. EAEC T6SS heterogeneity therefore represents a trade-off that solves a conflict between two opposing pressures: the need for ON cells to eliminate susceptible recipients, and the cost of provoking counterattacks from armed competitors.

- In a way, also the larvae data gives a hint. If I understand correctly, ON and OFF bacteria were mixed at a 1:1 ratio and the phenotype looks more like the OFF than the ON cells, potentially indicating that there is a disproportional disadvantage of phenotypic heterogeneity during infection?

Response : We agree that the phenotype observed in the Galleria model suggests that the benefits of heterogeneity may be context-dependent. We have now expanded the Discussion to explicitly mention this point and to propose that heterogeneity may confer advantages in interbacterial competition but not necessarily in host colonization.

Paragraph added in the Discussion:

This ON/OFF balance may, however, come at a cost in other biological contexts. In particular, while OFF cells may be advantageous in interbacterial competition by limiting exposure to retaliatory attacks, they may be less effective in host-associated environments where T6SS activity contributes to virulence or niche establishment. Indeed, our results showed that ON cells better colonize the wax moth model whereas OFF cells are less efficient. This context-dependent trade-off suggests that phenotypic heterogeneity may allow bacterial populations to balance competing selective pressures depending on the ecological conditions.

Out of curiosity, on the idea of phenotypic heterogeneity being an 'adaptive trait' and the described trade-off. Considering that T6SS activation via tit-for-tat is rather rare, do the authors consider P. aeruginosa a naturally occurring attacker during the evolutionary history of EAEC or do the authors have another more naturally co-occurring species with a T6SS in mind?

Response : We agree that *P. aeruginosa* may not be a natural competitor of EAEC. In this study, it is used as a well-characterized model of a retaliatory T6SS-positive bacterium. We have clarified this point in the manuscript.

Original sentence : However, when competing with the ~~defensive T6SS+ strain~~ *Pseudomonas aeruginosa* PAO1Δ*retS*, increasing the ON/OFF ratio led to higher EAEC mortality (Fig. 4b).

Revised sentence : However, when competing with the model retaliatory T6SS-positive competitor *Pseudomonas aeruginosa* PAO1Δ*retS* (i.e., a strain that constitutively expresses the H1-T6SS but only assembles it and fires upon attacks from competitors [48]), increasing the ON/OFF ratio led to higher EAEC mortality (Fig. 4b).
