# Supplementary material for: Phenotypic heterogeneity optimizes trade-offs during adaptive deployment of the type VI secretion system
Source: PLoS Biol. 2026 Jun 4;24(6):e3003838. doi: 10.1371/journal.pbio.3003838 (PMC13262931; doi:10.1371/journal.pbio.3003838)
Supplement: Attachment — Submitted filename: 2-Taillefer-Heterogeneity-ItemizedResponsesReviewers.pdf [file pbio.3003838.s015.pdf]

**Itemized responses to editor's and reviewer's comments.**

# Phenotypic heterogeneity optimizes trade-offs during adaptive deployment of the type VI secretion system

B. Taillefer, F. Schattenberg, T. Doan, S. Müller, E. Cascales

PBIOLOGY-D-25-03182

We thank the two reviewers for their positive assessment of the revised manuscript and for acknowledging that we carefully addressed their initial comments.

## Editorial comments

a) Please address the remaining comments from reviewer #1.

**Reviewer #1 :**
Comment : One aspect that is confusing in this figure: In panel b, the mortality of the prey strain W3110 decreases with higher T6SS activity in the attacker EAEC 17-2. This is counterintuitive, even when considering that (as mentioned in the manuscript) fewer EAEC 17-2 survive: In the 0% ON fraction, no W3110 are killed in Fig. 4b, so how are they killed here - by the PAO1? This should be discussed better.
Response: Figures 4b and 5b cannot be directly compared because they report the mortality of different strains in different competition settings. Figure 4b measures the mortality of enteroaggregative *E. coli* (EAEC, which carries a T6SS) during pairwise competition with *Pseudomonas aeruginosa* PAO1Δ*retS*, whereas Figure 5b measures the mortality of the T6SS-negative *E. coli* K-12 W3110 strain in a three-species competition assay. In the latter assay, W3110 is exposed not only to EAEC but also to P. *aeruginosa*. It was previously shown that *P. aeruginosa* can activate its H1-T6SS retaliatory response in response to envelope perturbations or cell-cell interactions, including those mediated by fimbriae or conjugative transfer. The residual mortality of W3110 observed when EAEC is in the 0% ON condition may therefore result from interactions between W3110 and *P. aeruginosa*. In addition, as the proportion of EAEC ON cells increases, reciprocal killing between EAEC and *P. aeruginosa* reduces the number of surviving EAEC cells, thereby limiting EAEC-mediated killing of W3110.

Comment: As a side note, I noticed that the y axis label in Fig. 4b reads "mortality (hours)", which is likely a mistake.

Response: It is not a mistake. As described in the Materials and Methods section, SGK assays measure the time required for surviving cells to reach mid-exponential phase (Tmid), which is used as a proxy for mortality. When more cells survive, growth resumes earlier and Tmid is lower; conversely, when killing is more efficient, growth resumes later and Tmid is higher. Thus, Tmid correlates with mortality and is expressed in hours.

b) You currently have 6 Figures, but the maximum allowable for our Short Report format is 4. Please reduce the number of Figures by combining some of them (several have a relatively simple structure, so this should be straightforward, without moving anything to the supplement).

The number of figures has been reduced from six to four by combining the previous Figures 1 and 2, and Figures 4 and 5. The text and figure legends have been updated accordingly.

c) Many thanks for supplying the underlying data. Please rename this supplementary data file ("1-Taillefer-Heterogeneity-RawData.xlsx") to "S1 Data.xlsx" and cite the location of the data clearly in all relevant main and supplementary Figure legends, e.g. "The data underlying this Figure can be found in S1 Data."

Corrected. The supplementary data file has been renamed "S1 Data.xlsx", and the relevant main and supplementary figure legends now indicate that the underlying data can be found in S1 Data.

e) I note that you mention the reviewers ("and the three anonymous reviewers for their constructive comments") in the Acknowledgements. While we appreciate the sentiment, this is against PLOS policy, so please could you remove this?

Corrected. The sentence mentioning the reviewers has been removed from the Acknowledgements section.

f) Please make any custom code available, either as a supplementary file or as part of your data deposition.

Not applicable. No custom code was generated or used for the analyses reported in this manuscript.

g) Please include the URLs of your funders in the Financial Disclosure statement.

The URLs of all funders have been included in the Financial Disclosure Statement in the submission system.
