# Supplementary material for: Phenotypic heterogeneity optimizes trade-offs during adaptive deployment of the type VI secretion system
Source: PLoS Biol. 2026 Jun 4;24(6):e3003838. doi: 10.1371/journal.pbio.3003838 (PMC13262931; doi:10.1371/journal.pbio.3003838)
Supplement: Peer review history

# Editor report: Decision Letter 0

**DOI:** 10.1371/journal.pbio.3003838.r001

**Authors:** Roland G Roberts

10 Oct 2025

Dear Dr Cascales,

Thank you for submitting your manuscript entitled “Phenotypic heterogeneity optimizes trade-offs during adaptive deployment of the type VI secretion system” for consideration as a Research Article by PLOS Biology.

Your manuscript has now been evaluated by the PLOS Biology editorial staff, as well as by an academic editor with relevant expertise, and I'm writing to let you know that we would like to send your submission out for external peer review.

IMPORTANT: We think that your manuscript would be better considered as a Short Report. No re-formatting is needed at this stage, but please select “Short Reports” as the article type when you upload your additional metadata (see next paragraph).

However, before we can send your manuscript to reviewers, we need you to complete your submission by providing the metadata that is required for full assessment. To this end, please login to Editorial Manager where you will find the paper in the 'Submissions Needing Revisions' folder on your homepage. Please click 'Revise Submission' from the Action Links and complete all additional questions in the submission questionnaire.

Once your full submission is complete, your paper will undergo a series of checks in preparation for peer review. After your manuscript has passed the checks it will be sent out for review. To provide the metadata for your submission, please Login to Editorial Manager (https://www.editorialmanager.com/pbiology) within two working days, i.e. by Oct 14 2025 11:59PM.

If your manuscript has been previously peer-reviewed at another journal, PLOS Biology is willing to work with those reviews in order to avoid re-starting the process. Submission of the previous reviews is entirely optional and our ability to use them effectively will depend on the willingness of the previous journal to confirm the content of the reports and share the reviewer identities. Please note that we reserve the right to invite additional reviewers if we consider that additional/independent reviewers are needed, although we aim to avoid this as far as possible. In our experience, working with previous reviews does save time.

If you would like us to consider previous reviewer reports, please edit your cover letter to let us know and include the name of the journal where the work was previously considered and the manuscript ID it was given. In addition, please upload a response to the reviews as a 'Prior Peer Review' file type, which should include the reports in full and a point-by-point reply detailing how you have or plan to address the reviewers' concerns.

During the process of completing your manuscript submission, you will be invited to opt-in to posting your pre-review manuscript as a bioRxiv preprint. Visit http://journals.plos.org/plosbiology/s/preprints for full details. If you consent to posting your current manuscript as a preprint, please upload a single Preprint PDF.

Feel free to email us at plosbiology@plos.org if you have any queries relating to your submission.

Kind regards,

Roli Roberts

Roland Roberts, PhD

Senior Editor

PLOS Biology

rroberts@plos.org

# Peer review: Decision Letter 1

**DOI:** 10.1371/journal.pbio.3003838.r002

**Authors:** Roland G Roberts

20 Nov 2025

Dear Dr Cascales,

Thank you for your patience while your manuscript "Phenotypic heterogeneity optimizes trade-offs during adaptive deployment of the type VI secretion system" was peer-reviewed at PLOS Biology. It has now been evaluated by the PLOS Biology editors, an Academic Editor with relevant expertise, and by three independent reviewers.

You'll see that reviewer #1 says the study is “simple, straightforward, and nicely presented.” However, s/he thinks that some of the claims of novelty are overstated, citing several papers that report similar instances of heterogeneity (though the current study still adds significant value), and his/her second major point requests further experimental evidence. Reviewer #2 says this is “a very cool finding and interesting to the field.” However, s/he identifies several missing controls and wants an improved statistical treatment, plus some presentational improvements. Reviewer #3 is very positive, calling the study “timely” and “of interest,” but also requesting some clarifications and better coverage of the literature.

In light of the reviews, which you will find at the end of this email, we would like to invite you to revise the work to thoroughly address the reviewers' reports.

Given the extent of revision needed, we cannot make a decision about publication until we have seen the revised manuscript and your response to the reviewers' comments. Your revised manuscript is likely to be sent for further evaluation by all or a subset of the reviewers.

In addition to these revisions, you will need to complete some formatting changes, which you will receive in a follow up email. A member of our team will be in touch with a set of requests shortly.

We expect to receive your revised manuscript within 3 months. Please email us (plosbiology@plos.org) if you have any questions or concerns, or would like to request an extension.

At this stage, your manuscript remains formally under active consideration at our journal; please notify us by email if you do not intend to submit a revision so that we may withdraw it.

**IMPORTANT - SUBMITTING YOUR REVISION**

Your revisions should address the specific points made by each reviewer. Please submit the following files along with your revised manuscript:

1. A 'Response to Reviewers' file - this should detail your responses to the editorial requests, present a point-by-point response to all of the reviewers' comments, and indicate the changes made to the manuscript.

*NOTE: In your point-by-point response to the reviewers, please provide the full context of each review. Do not selectively quote paragraphs or sentences to reply to. The entire set of reviewer comments should be present in full and each specific point should be responded to individually, point by point.

You should also cite any additional relevant literature that has been published since the original submission and mention any additional citations in your response.

2. In addition to a clean copy of the manuscript, please also upload a 'track-changes' version of your manuscript that specifies the edits made. This should be uploaded as a "Revised Article with Changes Highlighted" file type.

*Re-submission Checklist*

When you are ready to resubmit your revised manuscript, please refer to this re-submission checklist: https://plos.io/Biology_Checklist

To submit a revised version of your manuscript, please go to https://www.editorialmanager.com/pbiology/ and log in as an Author. Click the link labelled 'Submissions Needing Revision' where you will find your submission record.

Please make sure to read the following important policies and guidelines while preparing your revision:

*Published Peer Review*

Please note while forming your response, if your article is accepted, you may have the opportunity to make the peer review history publicly available. The record will include editor decision letters (with reviews) and your responses to reviewer comments. If eligible, we will contact you to opt in or out. Please see here for more details:

https://blogs.plos.org/plos/2019/05/plos-journals-now-open-for-published-peer-review/

*PLOS Data Policy*

Please note that as a condition of publication PLOS' data policy (http://journals.plos.org/plosbiology/s/data-availability) requires that you make available all data used to draw the conclusions arrived at in your manuscript. If you have not already done so, you must include any data used in your manuscript either in appropriate repositories, within the body of the manuscript, or as supporting information (N.B. this includes any numerical values that were used to generate graphs, histograms etc.). For an example see here: http://www.plosbiology.org/article/info%3Adoi%2F10.1371%2Fjournal.pbio.1001908#s5

*Blot and Gel Data Policy*

We require the original, uncropped and minimally adjusted images supporting all blot and gel results reported in an article's figures or Supporting Information files. We will require these files before a manuscript can be accepted so please prepare them now, if you have not already uploaded them. Please carefully read our guidelines for how to prepare and upload this data: https://journals.plos.org/plosbiology/s/figures#loc-blot-and-gel-reporting-requirements

*Protocols deposition*

To enhance the reproducibility of your results, we recommend that if applicable you deposit your laboratory protocols in protocols.io, where a protocol can be assigned its own identifier (DOI) such that it can be cited independently in the future. Additionally, PLOS ONE offers an option for publishing peer-reviewed Lab Protocol articles, which describe protocols hosted on protocols.io. Read more information on sharing protocols at https://plos.org/protocols?utm_medium=editorial-email&utm_source=authorletters&utm_campaign=protocols

Thank you again for your submission to our journal. We hope that our editorial process has been constructive thus far, and we welcome your feedback at any time. Please don't hesitate to contact us if you have any questions or comments.

Sincerely,

Roli Roberts

Roland Roberts, PhD

Senior Editor

PLOS Biology

rroberts@plos.org

------------------------------------

REVIEWERS' COMMENTS:

Reviewer #1:

The type VI secretion system (T6SS) is a nanoweapon commonly used in bacterial competition. Its regulation differs between bacteria, ranging from stochastic attacks to specific activation, such as the tit-for-tat mechanism where bacteria react to attacks from neighboring bacteria with retaliatory T6SS attack. Often, T6SS are only expressed and/or activated under specific conditions. T6SS activation is energetically costly and can provoke counterattacks. In line with this, T6SS were frequently lost during evolution. Considering these points, phenotypic heterogeneity within a population could be beneficial. This manuscript studies this question. It builds on an earlier publication by the Cascales group, in which they found a DNA methylation-based, iron-dependent epigenetic switch for the enteroaggregative Escherichia coli (EAEC) sci1 T6SS gene cluster (Brunet et al., PLOS Genetics (2011), doi 10.1371/journal.pgen.1002205). The current manuscript mainly uses microscopy to determine expression and assembly of the EAEC sci1 T6SS. For both aspects, the authors observe heterogeneity within populations, with T6SS states being stable for a few generations. The study is simple, straightforward, and nicely presented. However, I have concerns about some aspects of the claims and interpretation.

Major points:

1. As described above, heterogeneity of T6SS expression within clonal populations is conceptually not surprising. Heterogeneity for the assembly of the T6SS (using TssB fluorescent fusions) has been observed in many cases for different organisms. While most of these studies did not specifically investigate the heterogeneity, there are some which did. A study very directly addressing this point is Kerwien et al, PLOS Pathogens (2025), doi 10.1371/journal.ppat.1013356 (for Yersinia pseudotuberculosis), specific aspects are also shown in Lin et al, PNAS Nexus (2023), doi 10.1093/pnasnexus/pgad195 (for Vibrio fischeri, with a modeling focus) and Chen et al, Communication Biology (2025), doi 10.1038/s42003-025-08693-6 (for Yersinia enterocolitica, focusing on coregulation with other factors). The authors themselves mention several additional studies in the discussion (refs. 50-54). The manuscript under review is a valuable addition, as it additionally looks at expression specifically, the stability of heterogeneity across populations on the basis of the previously found epigenetic switch, and tries to show the role of heterogeneity in competition in a quantitative way. Nevertheless, the novelty claims in the abstract and several points in the manuscript do not appear justified in their current form.

2. Several aspects of Figure 4, the experiment aiming to show benefits of the heterogenous expression in specific conditions, are unclear. How do the authors get and analyze the 0-100% "ON" cell fractions? Why is even the 100% "ON" fraction killed less by P. aeruginosa than the WT, whose advantage should be precisely that it is killed less due to its heterogenous T6SS expression? To support the claim that the heterogenous expression enhances competitiveness in near-native conditions would require stronger results, including multi-species competition assay, as depicted in Fig. 5.

Minor points:

1. Line 82. Check sentence (expression is debated?).

2. Line 164. What is meant by "homogeneous ON state" in Fur box mutants? Fig. 3B shows T6SS+ rates of 45% (lower than WT) to 65% (higher, but far from 100%).

3. Lines 182-186. Is it really clear from the data (e.g. Fig. S5) that the overexpression strain (F2) also more frequently activates the T6SS? If not, the statement should be specified.

4. The "OFF" population in Fig. 2a, right panel, also seems to express the T6SS heterogeneously (albeit in a lower percentage of cells).

Reviewer #2:

Comments to the authors:

In this manuscript the authors observe significant heterogeneity in T6SS expression in enteroaggregative E.coli (EAEC). The expression of this T6SS locus has previously been shown to be under the regulatory control of the transcription factor Fur and the Dam methylase. Using nifty and innovative approaches, the authors investigate possible evolutionary reasons for heterogeneous expression. They find that heterogeneity arises from methylation dependent epigenetic switches and that although expression of T6SS does not come at a fitness cost, provoking tit-for-tat retaliation from bystander bacteria does. Cells not expressing the complete T6SS system are still protected by toxin delivery from kin-bacteria, suggesting that heterogeneous expression of the completeT6SS could be a bet-hedging strategy where a subpopulation of ON cells will protect the colony from attackers, while OFF cells are protected from tit-for-tat mediated attacks.

This is a very cool finding and interesting to the field. The manuscript is well written and adds our understanding on bacterial warfare and the burdens of toxin delivery on bacteria. However, there are a few concerns that would require attention before the manuscript could be acceptable for publication. Mainly, some controls and statistical analyses are lacking, and some textual /illustrational modification would also benefit the manuscript. See the main and minor comments below for more detail.

Main comments:

1. One of the main arguments of the above theory is that the OFF population is protected from kin-delivery through expression of the TliI antitoxin from a different promoter inside the T6SS locus. The authors test this by competing WT, ON (F2) and deltasci1 inhibitors against WT and OFF (G123) targets. However, in Fig S2 only WT targets are shown. The data with the OFF targets mentioned in the text should be included in the actual figure.

It is also not clear why these experiments where performed at a 1:4 ratio, rather than the 4:1 ration used in Fig. 3C. Using a lower ratio could falsely indicate that there is protection against kin-bacteria, in particular as there is no control included where inhibition actually occurs. This data is vital for the model to work. Therefore, the experiment should be repeated at a 4:1 ratio, including a control showing that indeed T6SS mediated killing does occur in this context. For proper statistical analysis and to align better with the rest of the experimnets, more than 2 bioreps should be used.

2. The second important point that the authors make is that the ON/OFF state is determined by a Dam-methylation dependent epigenetic switch. I agree that this is likely, but the data in figure S4C lacks statistical analysis, making it difficult to assess whether the sorted ON colonies indeed stay ON etc. Anova with posthoc test would tell if this is indeed the case. Also, discussion about the switch-rates for ON/OFF would be interesting and could be calculated on the same data.

3. In general, statistics seem to be missing in many figures: Fig 3b, Fig 4abc, Fig S7d. Please add the appropriate statistical analyses to the figures. In addition, please provide the raw-data for the experiments as a supplementary excel file.

4. The assay used for enumerating target survival in figs 4ab and S7b is somewhat untraditional. It is unclear how the authors quantify survival from the hours it takes for the culture to grow to a certain OD600, when no standard curve is shown. Please include a standard curve and maybe an overview figure for this method to make it more clear.

4. There seems to be a loss in virulence/colonization ability for the OFF mutants in the Galleria mellonella assay. This should at least be mentioned in the results/discussion as this is rather cool.

Minor comments:

1. Skip the B-GFP, C-GFP-K abbreviations throughout the manuscript. They are mainly used in the figures and just confuse the reader. There is plenty of space to write out the full name of the fusion in the figures, which would make the manuscript much easier to read.

2. Figure 2 legend: "Short, medium and long: frequencies after ~5, ~15 and ~20 generations,

622 respectively." Write the number of generations in the graph instead of long, medium, short. The terms long, medium, short are unclear and could be reffering to e.g. cell length instead.

3. Line 45. "…ts.." - meaning not clear. Should this say T6SS?

4. Line 111: "These observations suggest that heterogeneity occurs at both the transcriptional/translational level (expression/production vs.no expression/production) and the post-translational level (assembly vs. no assembly)"-This statement requires some controls: i.e. a strain lacking T6SS and a strain unable to assemble T6SS to show that what you observe in the images are indeed T6SS foci and lack of the same.

5. Line 133: Individual founder cells gave rise to homogeneous OFF,homogeneous ON, or heterogeneous microcolonies. - How were the different populations determined? What cut-offs of fluorescence was used? Maybe illustrate this in the histograms of Figure 1ab.

6. Line 134: Homogeneous ON and heterogeneous colonies were more frequent than homogeneous OFF colonies, resulting in a population distribution similar to that observed in liquid culture (CV > 0.20; Fig. 2b) - Data for quantification of ON and OFF colonies is not shown. Etiher remove this statement or add the data.

7. Line 136: "The overall frequency of T6SS⁺ cells, which was lower in microfluidics, likely due to slower growth under reduced oxygen availability compared with liquid conditions" -Odd sentence. Please rephrase.

8. Line 206. Please state why you used a strain with a deltaretS. This might not be clear for the non-expert readers.

9. "Fig. 3c shows that the T6SS activity correlated with the proportion of T6SS⁺ cells: G1 and G2 mutants exhibited reduced killing, while G3 and G123mutants were inactive under inducing conditions (Fig. 3c)." .Not shown in Fig 3c. G1 and G3 look identical. G2 looks like wt and G123 looks OFF. Please rephrase or show quantitative data if you want to claim this.

Also, please include an illustration or text in the figure (not only in the legend) explaining how the color change illustrates viability of targets.

10. There is something odd with the peaks in figure S6ab. The peaks are either cut off at random values or there were very few data points recorded for e.g. G123 and G3 in a and G1, G2, G3 and G123 in b. Please fix this.

Reviewer #3:

In this manuscript, Taillefer and colleagues focus on phenotypic heterogeneity of the T6SS. Although I would say that this phenomenon could be observed in microscopy images of existing studies already, I agree that there has been little work entirely dedicated to phenotypic heterogeneity of the T6SS. This manuscript is very timely. The authors use an array of techniques, ranging from sophisticated microscopy to cell sorting, genetics, in vivo infection experiments, and bacterial competition assays to precisely quantify the ratio of cells with and without a T6SS sheath in a population, demonstrate that ON and OFF states are reversible, decipher the mechanism that controls the ON and OFF state, and assess the role of phenotypic heterogeneity for killing others and for being attacked. These findings shed light on a topic that has received little attention so far in studies on T6SS activity and provides important data in the ongoing debate on the costs and benefits of the T6SS. I therefore see the results of broad interest to the fields of the T6SS, phenotypic heterogeneity, and the evolution of trade-offs.

In my opinion, the experimental work is done very thoroughly, the manuscript is clearly written and the data is well presented.

I have a few comments that I would please ask the authors to consider during their revision.

Major comments

1. Use of the term "homogeneous ON" and "homogeneous OFF". Could the authors at least clarify, please. As I understand, S3 Fig. shows examples of what is referred to as "homogeneous ON" and "homogeneous OFF" in l. 133. I see the strong difference between the two, I just think it is important that the ones considered as ON are actually not all ON but some do not have the sheath, isn't it? Also among the ones that are OFF, there seems to be at least one cell that is ON, or am I missing something here? This makes me very generally wondering about the idea of the competition experiments, where cells are mixed at a certain ON and OFF ratio. How much do we know about the actual ratio of bacteria with and without a functional T6SS in these spots? Isn't "ON" rather a population of bacteria with a high percentage of bacteria with a T6SS formed and "OFF" rather a population of bacteria with a low percentage of bacteria with a T6SS formed? If so, are the terms 'ON' and 'OFF' and 'homogeneous' potentially slightly misleading? In the context of this comment, it might also be worth reconsidering the graphical depiction in Fig. 5.

2. I encourage the authors to expand a bit more on existing data in other species of relevance to T6SS phenotypic heterogeneity (e.g. PMID: 40991669, PMID: 40819180). I understand that the work in the manuscript is on EAEC, do the authors expect such heterogeneity in the T6SS across species? Does existing T6SS microscopy data suggest similar phenotypes in other species?

Minor comments

l. 28 "deliver" should probably read "delivers"

l. 45 typo "its"?

l. 65 just a thought and up to the authors, I personally struggle a bit with „within a genetically identical population". Aren't bacterial populations (even an overnight culture of the WT strain) rather genetically diverse? I guess they can be genetically diverse without being a problem for the story, as long as the genetic differences are not relevant for the particular trait of interest, here T6SS activity.

Fig. 1a, I might have missed this, is a brightfield image provided? How else do the authors conclude on the first of the three subpopulations (cells that do not produce TssB-sfGFP). Did the authors somehow validate their method to reliably detect cells with the TssB tube?

Might be worth checking the figure legend again, I wasn't sure about the references to left and right.

Fig. 3c, could the authors please indicate if these are representative images of multiple replicates?

Fig. 4 and respective text (l. 200f), I have multiple comments or thoughts here:

- I would be interested in what the ticks on the x-axis refer to, I guess it is steps of 20% but wasn't sure. Could the actually numbers be added in addition to the triangle?

- How do the authors explain that the WT resembles a phenotype of ON in Fig. 4b? isn't this unexpected given the idea of phenotypic heterogeneity and expected 60% ON based on the data in Fig. 1? Also, how come that the WT resembles phenotypic data of different on/off ratios in Fig. 4a and 4b?

- Just a side note, isn't it really interesting that there seems to be a linear relationship between the on/off ratio and killing others, and a non-linear relationship between the on/off ratio and getting killed by P. aeruginosa? In other words, there are certain ratios that could provide a disproportionally higher gain from killing others than becoming costly when mixed with an attacker that does tit-for-tat.

- In a way, also the larvae data gives a hint. If I understand correctly, ON and OFF bacteria were mixed at a 1:1 ratio and the phenotype looks more like the OFF than the ON cells, potentially indicating that there is a disproportional disadvantage of phenotypic heterogeneity during infection?

Out of curiosity, on the idea of phenotypic heterogeneity being an 'adaptive trait' and the described trade-off. Considering that T6SS activation via tit-for-tat is rather rare, do the authors consider P. aeruginosa a naturally occurring attacker during the evolutionary history of EAEC or do the authors have another more naturally co-occurring species with a T6SS in mind?

# Author comment: Author response to Decision Letter 2

**DOI:** 10.1371/journal.pbio.3003838.r003

14 Apr 2026

Attachment Submitted filename: 1-Taillefer-Heterogeneity-ItemizedResponsesReviewers.pdf

# Peer review: Decision Letter 2

**DOI:** 10.1371/journal.pbio.3003838.r004

**Authors:** Roland G Roberts

16 May 2026

Dear Dr Cascales,

Thank you for your patience while we considered your revised manuscript "Phenotypic heterogeneity optimizes trade-offs during adaptive deployment of the type VI secretion system" for publication as a Short Report at PLOS Biology. This revised version of your manuscript has been evaluated by the PLOS Biology editors, the Academic Editor and two of the original reviewers.

Based on the reviews and our Academic Editor's assessment of your revision, we are likely to accept this manuscript for publication, provided you satisfactorily address the remaining points raised by the reviewers and the following data and other policy-related requests.

IMPORTANT - please attend to the following:

a) Please address the remaining comments from reviewer #1.

b) You currently have 6 Figures, but the maximum allowable for our Short Report format is 4. Please reduce the number of Figures by combining some of them (several have a relatively simple structure, so this should be straightforward, without moving anything to the supplement).

c) Many thanks for supplying the underlying data. Please rename this supplementary data file ("1-Taillefer-Heterogeneity-RawData.xlsx") to "S1 Data.xlsx" and cite the location of the data clearly in all relevant main and supplementary Figure legends, e.g. “The data underlying this Figure can be found in S1 Data.”

e) I note that you mention the reviewers ("and the three anonymous reviewers for their constructive comments") in the Acknowledgements. While we appreciate the sentiment, this is against PLOS policy, so please could you remove this?

f) Please make any custom code available, either as a supplementary file or as part of your data deposition.

g) Please include the URLs of your funders in the Financial Disclosure statement.

As you address these items, please take this last chance to review your reference list to ensure that it is complete and correct. If you have cited papers that have been retracted, please include the rationale for doing so in the manuscript text, or remove these references and replace them with relevant current references. Any changes to the reference list should be mentioned in the cover letter that accompanies your revised manuscript.

In addition to these revisions, you may need to complete some formatting changes, which you will receive in a follow up email. A member of our team will be in touch with a set of requests shortly. If you do not receive a separate email within a few days, please assume that checks have been completed, and no additional changes are required.

We expect to receive your revised manuscript within two weeks.

To submit your revision, please go to https://www.editorialmanager.com/pbiology/ and log in as an Author. Click the link labelled 'Submissions Needing Revision' to find your submission record. Your revised submission must include the following:

- a cover letter that should detail your responses to any editorial requests, if applicable, and whether changes have been made to the reference list

- a Response to Reviewers file that provides a detailed response to the reviewers' comments (if applicable, if not applicable please do not delete your existing 'Response to Reviewers' file.)

- a track-changes file indicating any changes that you have made to the manuscript.

NOTE: If Supporting Information files are included with your article, note that these are not copyedited and will be published as they are submitted. Please ensure that these files are legible and of high quality (at least 300 dpi) in an easily accessible file format. For this reason, please be aware that any references listed in an SI file will not be indexed. For more information, see our Supporting Information guidelines:

https://journals.plos.org/plosbiology/s/supporting-information

*Published Peer Review History*

Please note that you may have the opportunity to make the peer review history publicly available. The record will include editor decision letters (with reviews) and your responses to reviewer comments. If eligible, we will contact you to opt in or out. Please see here for more details:

https://plos.org/published-peer-review-history/

*Press*

Should you, your institution's press office or the journal office choose to press release your paper, please ensure you have opted out of Early Article Posting on the submission form. We ask that you notify us as soon as possible if you or your institution is planning to press release the article.

*Protocols deposition*

To enhance the reproducibility of your results, we recommend that if applicable you deposit your laboratory protocols in protocols.io, where a protocol can be assigned its own identifier (DOI) such that it can be cited independently in the future. Additionally, PLOS ONE offers an option for publishing peer-reviewed Lab Protocol articles, which describe protocols hosted on protocols.io. Read more information on sharing protocols at https://plos.org/protocols?utm_medium=editorial-email&utm_source=authorletters&utm_campaign=protocols

Please do not hesitate to contact me should you have any questions.

Sincerely,

Roli Roberts

Roland Roberts, PhD

Senior Editor

rroberts@plos.org

PLOS Biology

------------------------------------------------------------------------

CODE POLICY

Per journal policy, if you have generated any custom code during the course of this investigation, please make it available without restrictions. Please ensure that the code is sufficiently well documented and reusable, and that your Data Statement in the Editorial Manager submission system accurately describes where your code can be found. More information on our Code Policy, what and how to share can be found here: https://journals.plos.org/plosbiology/s/code-availability

Please note that we cannot accept sole deposition of code in GitHub, as this could be changed after publication. However, you can archive this version of your publicly available GitHub code to Zenodo. Once you do this, it will generate a DOI number, which you will need to provide in the Data Accessibility Statement (you are welcome to also provide the GitHub access information). See the process for doing this here: https://docs.github.com/en/repositories/archiving-a-github-repository/referencing-and-citing-content

------------------------------------------------------------------------

DATA NOT SHOWN?

- Please note that per journal policy, we do not allow the mention of "data not shown", "personal communication", "manuscript in preparation" or other references to data that is not publicly available or contained within this manuscript. Please either remove mention of these data or provide figures presenting the results and the data underlying the figure(s).

------------------------------------------------------------------------

REVIEWERS' COMMENTS:

Reviewer #1:

The authors have addressed my comments (and, as far as I can see, the comments of the other reviewers) not only by clarifying statements and figures, but also by including additional experiments. I appreciate this additional work, especially the inclusion of the new figure 5 showing the three-species competition assay, which truly strengthens the manuscript.

One aspect that is confusing in this figure: In panel b, the mortality of the prey strain W3110 decreases with higher T6SS activity in the attacker EAEC 17-2. This is counterintuitive, even when considering that (as mentioned in the manuscript) fewer EAEC 17-2 survive: In the 0% ON fraction, no W3110 are killed in Fig. 4b, so how are they killed here - by the PAO1? This should be discussed better.

As a side note, I noticed that the y axis label in Fig. 4b reads "mortality (hours)", which is likely a mistake.

Reviewer #2:

The authors have satisfactorily addressed all of my concerns and have included substantial new analyses and additional data that significantly strengthen the manuscript. The revisions improve the clarity, rigor, and overall impact of the study, and I now consider the manuscript suitable for publication.

# Author comment: Author response to Decision Letter 3

**DOI:** 10.1371/journal.pbio.3003838.r005

20 May 2026

Attachment Submitted filename: 2-Taillefer-Heterogeneity-ItemizedResponsesReviewers.pdf

# Editor report: Decision Letter 3

**DOI:** 10.1371/journal.pbio.3003838.r006

**Authors:** Roland G Roberts

21 May 2026

Dear Dr Cascales,

Thank you for the submission of your revised Short Report "Phenotypic heterogeneity optimizes trade-offs during adaptive deployment of the type VI secretion system" for publication in PLOS Biology. On behalf of my colleagues and the Academic Editor, Victor Sourjik, I'm pleased to say that we can in principle accept your manuscript for publication, provided you address any remaining formatting and reporting issues. These will be detailed in an email you should receive within 2-3 business days from our colleagues in the journal operations team; no action is required from you until then. Please note that we will not be able to formally accept your manuscript and schedule it for publication until you have completed any requested changes.

Please take a minute to log into Editorial Manager at http://www.editorialmanager.com/pbiology/, click the "Update My Information" link at the top of the page, and update your user information to ensure an efficient production process.

PRESS: We frequently collaborate with press offices. If your institution or institutions have a press office, please notify them about your upcoming paper at this point, to enable them to help maximise its impact. If the press office is planning to promote your findings, we would be grateful if they could coordinate with biologypress@plos.org. If you have previously opted in to the early version process, we ask that you notify us immediately of any press plans so that we may opt out on your behalf.

We also ask that you take this opportunity to read our Embargo Policy regarding the discussion, promotion and media coverage of work that is yet to be published by PLOS. As your manuscript is not yet published, it is bound by the conditions of our Embargo Policy. Please be aware that this policy is in place both to ensure that any press coverage of your article is fully substantiated and to provide a direct link between such coverage and the published work. For full details of our Embargo Policy, please visit http://www.plos.org/about/media-inquiries/embargo-policy/.

Thank you again for choosing PLOS Biology for publication and supporting Open Access publishing. We look forward to publishing your study.

Sincerely,

Roli Roberts

Roland G Roberts, PhD, PhD

Senior Editor

PLOS Biology

rroberts@plos.org
